# The Neural and Computational Architecture of Feedback Dynamics in Mouse Cortex during Stimulus Report

**DOI:** 10.1523/ENEURO.0191-24.2024

**Published:** 2024-09-24

**Authors:** Simone Ciceri, Matthijs N. Oude Lohuis, Vivi Rottschäfer, Cyriel M. A. Pennartz, Daniele Avitabile, Simon van Gaal, Umberto Olcese

**Affiliations:** ^1^Institute for Theoretical Physics, Utrecht University, Utrecht 3584CC, Netherlands; ^2^Cognitive and Systems Neuroscience, Swammerdam Institute for Life Sciences, University of Amsterdam, Amsterdam 1098XH, Netherlands; ^3^Research Priority Area Brain and Cognition, University of Amsterdam, Amsterdam 1098XH, Netherlands; ^4^Mathematical Institute, Leiden University, Leiden 2333CA, Netherlands; ^5^Korteweg-de Vries Institute for Mathematics, University of Amsterdam, Amsterdam 1098XG, Netherlands; ^6^Amsterdam Center for Dynamics and Computation, Mathematics Department, Vrije Universiteit Amsterdam, Amsterdam 1081HV, Netherlands; ^7^Mathneuro Team, Inria Centre at Université Côte d’Azur, Sophia Antipolis 06902, France; ^8^Amsterdam Neuroscience, Systems and Network Neuroscience, Amsterdam 1081HV, Netherlands; ^9^Department of Psychology, University of Amsterdam, Amsterdam 1018WT, Netherlands

**Keywords:** computational neuroscience, consciousness, perception, report-related activity

## Abstract

Conscious reportability of visual input is associated with a bimodal neural response in the primary visual cortex (V1): an early-latency response coupled to stimulus features and a late-latency response coupled to stimulus report or detection. This late wave of activity, central to major theories of consciousness, is thought to be driven by the prefrontal cortex (PFC), responsible for “igniting” it. Here we analyzed two electrophysiological studies in mice performing different stimulus detection tasks and characterized neural activity profiles in three key cortical regions: V1, posterior parietal cortex (PPC), and PFC. We then developed a minimal network model, constrained by known connectivity between these regions, reproducing the spatiotemporal propagation of visual- and report-related activity. Remarkably, while PFC was indeed necessary to generate report-related activity in V1, this occurred only through the mediation of PPC. PPC, and not PFC, had the final veto in enabling the report-related late wave of V1 activity.

## Significance Statement

A long-standing objective in the study of consciousness is to characterize the neural signatures of perceived compared with unperceived sensory stimuli. In particular, the role of different cortical areas in generating consciousness is an active debate. Here we combined electrophysiological and modeling studies to investigate the mechanistic role of individual connections between brain regions in the generation of the activity patterns observed during conscious report. We found that, while frontal areas initiate report-related activity, the parietal cortex acts as a gate to determine whether such activity will propagate back to the visual cortex. This division of labor between prefrontal and parietal cortices is unexplained by current theoretical models of consciousness and will impact mechanistic neural models of conscious report.

## Introduction

A long-standing objective in the investigation of the neural mechanisms of consciousness is to characterize the signatures of perceived versus nonperceived stimuli at the level of neurons and their interactions (Aru et al., 2012; Koch et al., 2016). A major step forward regarding this has been the discovery of a bimodal neural response in the primary visual cortex: an early-latency response coupled to stimulus presentation and a late-latency response that is only observed when agents report a stimulus (Supèr et al., 2001; Del Cul et al., 2007; van Vugt et al., 2018). Despite ongoing debates on the functional significance of this late-latency activity (Koch et al., 2016; Sergent et al., 2021; Hatamimajoumerd et al., 2022), there is agreement that it strongly correlates with explicit report across several species, e.g., primates (Supèr et al., 2001; van Vugt et al., 2018), ferrets (Yin et al., 2020), and mice (Allen et al., 2017; Steinmetz et al., 2019; Oude Lohuis et al., 2022c).

The origin of the late, report-related activity has been pinpointed to frontal areas. In several species, correlates of sensory detection behavior first originate in the prefrontal areas and only later appear in the association and sensory areas (Del Cul et al., 2007; van Vugt et al., 2018; Steinmetz et al., 2019; Yin et al., 2020). A recent study even demonstrated that activity in a secondary motor area (in mice considered to be part of the prefrontal cortex; Le Merre et al., 2021) is necessary for this late activity to emerge in mice (Allen et al., 2017). However, it is currently not understood how late, report-related activity reaches sensory regions and whether, besides originating in the prefrontal regions, it is also shaped by other cortical regions, and if so how. For instance, it is debated whether prefrontal regions directly trigger late, report-related activity in primary sensory cortices or whether this is (also) mechanistically driven by intermediate regions, such as association areas in the parietal and temporal lobes (Fahrenfort et al., 2008; Quiroga et al., 2008; Fisch et al., 2009; van Vugt et al., 2018). Addressing this question is important to better characterize how patterns of cortical activity that have been linked to conscious report are generated and propagate through the cortical regions and is consequential for arbitrating between major theories of consciousness (Seth and Bayne, 2022; COGITATE Consortium et al., 2023).

Nevertheless, it is currently unfeasible to fully dissect the circuit-level architecture underlying the origin and propagation of neural activity, and although options (chiefly optogenetics) are available to modulate individual cortical areas (Oude Lohuis et al., 2022a,b,c), this approach is unsuitable to causally manipulate individual connections between regions. Indeed, projection-specific optogenetic inactivation is only moderately effective on synaptic terminals or has relatively low temporal dynamics (Rost et al., 2022). The alternative approach of silencing the activity of feedback-projecting neurons, while achieving high efficacy and fast temporal specificity, inevitably modifies the activity of source cortical regions as well (Tervo et al., 2016; Huh et al., 2018). To overcome these limitations, we developed a minimal model of neural dynamics (Chaudhuri et al., 2015; Joglekar et al., 2018), testing the contribution of individual feedback pathways in report-related activity. Compared with previous human studies following a similar approach for studying report-related activity (Dehaene et al., 2003; Castro et al., 2020; Alilović et al., 2023; Canales-Johnson et al., 2023), we leveraged the recent availability of functional and structural data in mice (Harris et al., 2019; Steinmetz et al., 2019; Oude Lohuis et al., 2022c) to develop a computational model with anatomically faithful connectivity strengths between regions and capable of reproducing patterns of spiking activity that we observed in mice performing perceptual tasks. Both the model and neural analyses focused on mouse primary visual cortex (V1), posterior parietal cortex (PPC), and prefrontal cortex (PFC) to test to what extent PFC is necessary to generate report-related activity in V1 and what the role of association areas (e.g., PPC) is.

## Materials and Methods

### Experimental design

#### Collection and analysis of in vivo recordings

The model we developed (see below, Model description) was qualitatively fitted on data previously collected in vivo in two different sets of experiments that have been reported in, respectively, Oude Lohuis et al. (2022b,c, 2024) and Steinmetz et al. (2019). The experimental procedures performed to collect the data are only summarized here and are described more extensively in Oude Lohuis et al. (2022b,c, 2024) and Steinmetz et al. (2019). For the latter dataset, all raw data and metadata are publicly available in a published repository (see below for further details). However, this is not the case for the former dataset, as not all recordings have yet been made publicly available, and data is still only available from the authors upon reasonable request. For this reason, we will here mainly focus on describing experimental procedures related to the former set of experiments (Oude Lohuis et al., 2022b,c, 2024), so that all relevant details about yet-to-be-published experimental recordings are available.

#### Experimental subjects

All animal experiments followed the relevant national and institutional regulations. Experimental procedures were approved by the Dutch National Commission for Animal Experiments and by the Animal Welfare Body of the University of Amsterdam. The data presented here was collected from 17 male mice, obtained from two transgenic mouse lines: PVcre [B6;129P2-Pvalbtm1(cre)Arbr/J, RRID: IMSR_JAX:008069] and F1 offspring of this PVcre line and Ai9-TdTomato cre reporter mice [Gt(ROSA)26Sortm9(CAG-tdTomato)Hze RRID: ISMR_JAX 007909]. Mice were group-housed under a reversed day–night schedule (lights on at 20:00 and off at 8:00) and all experimental procedures were done in the dark period. Temperature was kept between 19.5 and 23.5°C, and humidity between 45 and 65%. During behavioral training (starting when mice were ∼8 week old), mice were kept under a water restriction regime. Their minimum weight was kept above 85% of their average weight between postnatal day (P) 60 and P90. Mice were normally trained 5 d/week and generally obtained all their daily liquids in the form of rewards during task performance. A supplement was delivered when the amount of liquid obtained during the task was below a minimum of 0.025 ml/g body weight per day. The same amount was provided during weekends. Mice received *ad libitum* food.

#### Surgical procedures

**Figure 1.**
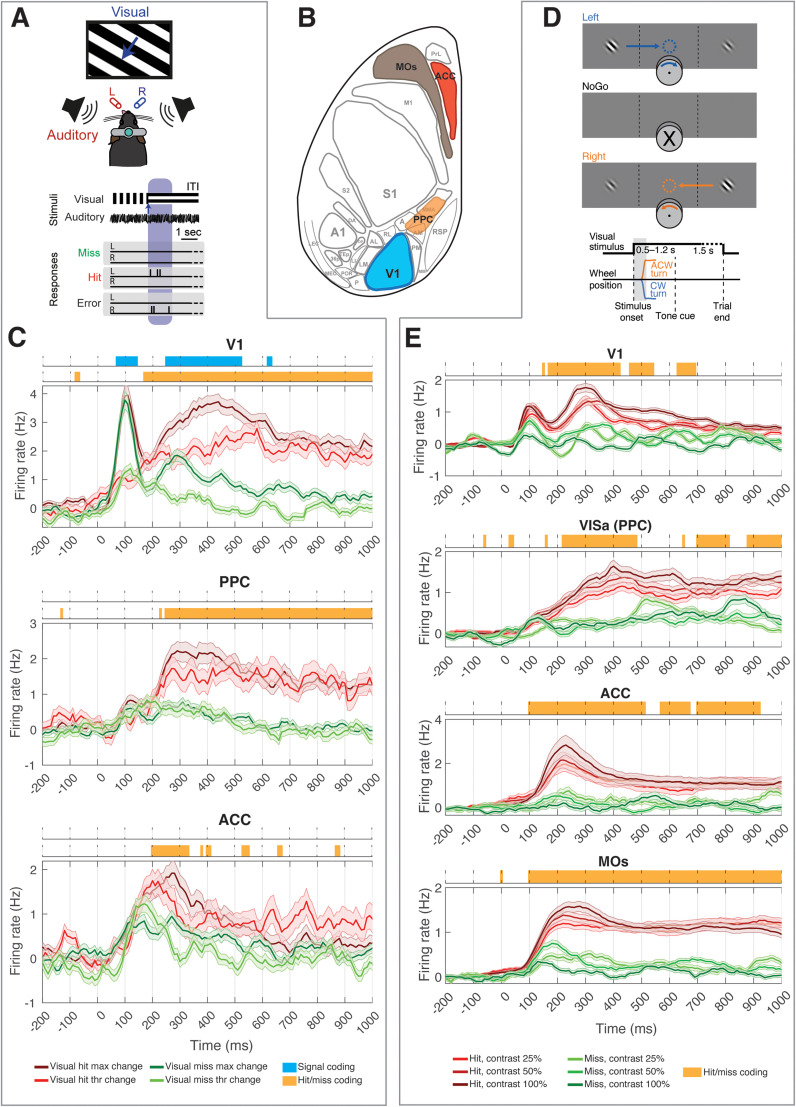
Neuronal correlates of perceptual decision making in the mouse cortex. ***A***, Top, Schematic of the experimental configuration of the audiovisual change detection paradigm for head-fixed mice. Bottom, Timeline of the audiovisual change detection task, indicating task contingencies for a visual change trial. Modified from Oude Lohuis et al. (2022b,c). ***B***, Schematic representation of the relevant cortical areas represented on a flattened cortical surface. Acronyms are used for the major subdivision of the dorsal cortex following standard nomenclature (Wang and Burkhalter, 2007; Wang et al., 2012; Steinmetz et al., 2019). Highlighted in color are the areas from which data was analyzed. V1, primary visual cortex; PPC, posterior parietal cortex; ACC, anterior cingulate cortex. MOs, Supplementary motor cortex. ***C***, Baseline-corrected average PSTHs recorded in (from top to bottom) V1, PPC, and ACC following a change in the orientation of the presented drifting grating. Red, hits; green, misses. Dark colors indicate max visual change (highest saliency), while light colors indicate threshold visual change (low saliency). Shaded areas indicate the standard error of the mean. Color bars on top of individual panels indicate time bins in which significant differences (*p* < 0.05, permutation-based test, FDR-corrected) were found between responses to, respectively, sensory stimuli with a difference salience (blue) or hit/miss trials (orange). See also Extended Data Figure 1-1. ***D***, Outline (top) and timeline (bottom) of the contrast discrimination task, in which mice had to rotate a wheel to bring the Gabor patch with the highest contrast toward the center of the field of view. CW, clockwise; ACW, anticlockwise. Modified from Steinmetz et al. (2019). ***E***, Same as ***C***, but computed as a function of the difference in contrast between the stimulus presented in the contralateral field of view with respect to the recorded hemisphere (which was always the highest-contrast stimulus) and the stimulus presented ipsilaterally. The color darkness indicates the contrast difference. Statistical differences were computed as in panel ***C***. Note that no difference between responses to sensory stimuli with different contrasts was observed. See also Extended Data Figure 1-1.

At the start of experimental procedures, mice were implanted with a headbar to allow head fixation in the experimental setup. Approximately 3 weeks before electrophysiological recordings, a subset of mice received an injection of an adeno-associated virus mediating the Cre-dependent expression of ChR2 in parvalbumin-positive interneurons; the injection was performed, in separate sets of mice, in either V1 or PPC. Data collected during optogenetic interventions was not utilized for the analyses presented in this study. The day before the start of extracellular recordings, small craniotomies (∼200 μm in diameter) were made over the cortical areas of interest using a dental drill. The cortical regions [V1, PPC, and anterior cingulate cortex (ACC) for this study] were identified either via stereotactic coordinates or via intrinsic optical signal imaging (Fig. 1*A*). Details about all surgical procedures can be found in Oude Lohuis et al. (2022b,c, 2024).

10.1523/ENEURO.0191-24.2024.f1-1 Figure 1-1 **(A)** Same as Fig. 1C, but including neuronal responses during false alarms (grey). Neuronal responses during error trials (licks toward the wrong side) are not shown in view of their rare occurrence. For visualization purposes, hit and miss trials are only shown for max visual change trials. **(B)** Same as Fig. 1E, but including neuronal responses during false alarms (grey) and during errors (black), the latter indicating responses towards the lowest-contrast visual stimulus. For visualization purposes, hit and miss trials are only shown for max visual change trials. Download Figure 1-1, TIF file.

#### Behavioral task and sensory stimuli

Mice were trained, over the course of several weeks, to perform an audio-visual change detection task (Fig. 1*A*).Visual stimuli were drifting square-wave gratings (temporal frequency, 1.5 Hz; spatial frequency, 0.08 cycles per degree; contrast, 70%; gamma-corrected), presented over the full screen (18.5 inch monitor, 60 Hz refresh rate). Gratings were continuously presented at a distance of ∼21 cm from the eyes. In a subset of trials (visual change trials), the orientation of the drifting grating was instantaneously changed. The degree of orientation change determined the visual saliency, which was set, based on the properties of the psychometric curve of individual mice, to a value corresponding to a threshold or max change (detection threshold and 90°, respectively). Mice were trained to respond to a visual change by licking to one reward port (left or right, counterbalanced across mice) and received 5–8 μl of liquid reward (infant formula) upon a correct response. Visual stimuli were the subject of analysis in the current manuscript, and a detailed description can be found in Oude Lohuis et al. (2022b,c). Correct responses to an auditory changes corresponded to licks toward the port not rewarded for visual stimuli (counterbalanced across mice). Importantly, similar neuronal responses were obtained across the measured areas irrespective of the side to which the mice had to lick upon a visual change, as well as independently of whether mice were trained to only report visual but not auditory changes. A more in-depth account can be found in Oude Lohuis et al. (2022b,c, 2024).

#### Multi-area recordings: acquisition and pre-processing

Extracellular recordings were performed simultaneously in 2 or 3 cortical areas (V1, PPC, ACC, and A1 were targeted in different experimental sessions). Recordings were performed on a maximum of 4 consecutive days. Several types of NeuroNexus silicon probes were used (A1 × 32-Poly2–10mm-50s-177, A2 × 16-10mm-100-500-177, A4 × 8-5mm-100-200-177, A1 × 64-Poly2-6mm-23s-160). Neurophysiological signals were preamplified, bandpass filtered (0.1 Hz–9 kHz), and acquired at 32 kHz (a bandpass filter was set between 0.1 Hz and 9 kHz) with a Digital Lynx SX 128 channel system, via the acquisition software Cheetah 5.0 (Neuralynx). Spike detection and sorting were performed using the Klusta (version 3.0.16) and Phy (version 1.0.9) software packages. For more details about acquisition and preprocessing, refer to Oude Lohuis et al. (2022b,c, 2024).

#### Histology

At the end of experiments, mice were perfused in 4% PFA in PBS and their brains were recovered for histological reconstruction meant to verify the correct placement of silicon probes in V1, PPC, and ACC.

#### Data availability

All the data used for the analyses presented in Figure 1 will be shared by the corresponding authors upon request.

This paper also analyzes existing, publicly available data from a study previously published by an independent lab (Steinmetz et al., 2019). This is available at https://doi.org/10.6084/m9.figshare.11274968.

#### Model description

**Figure 2.**
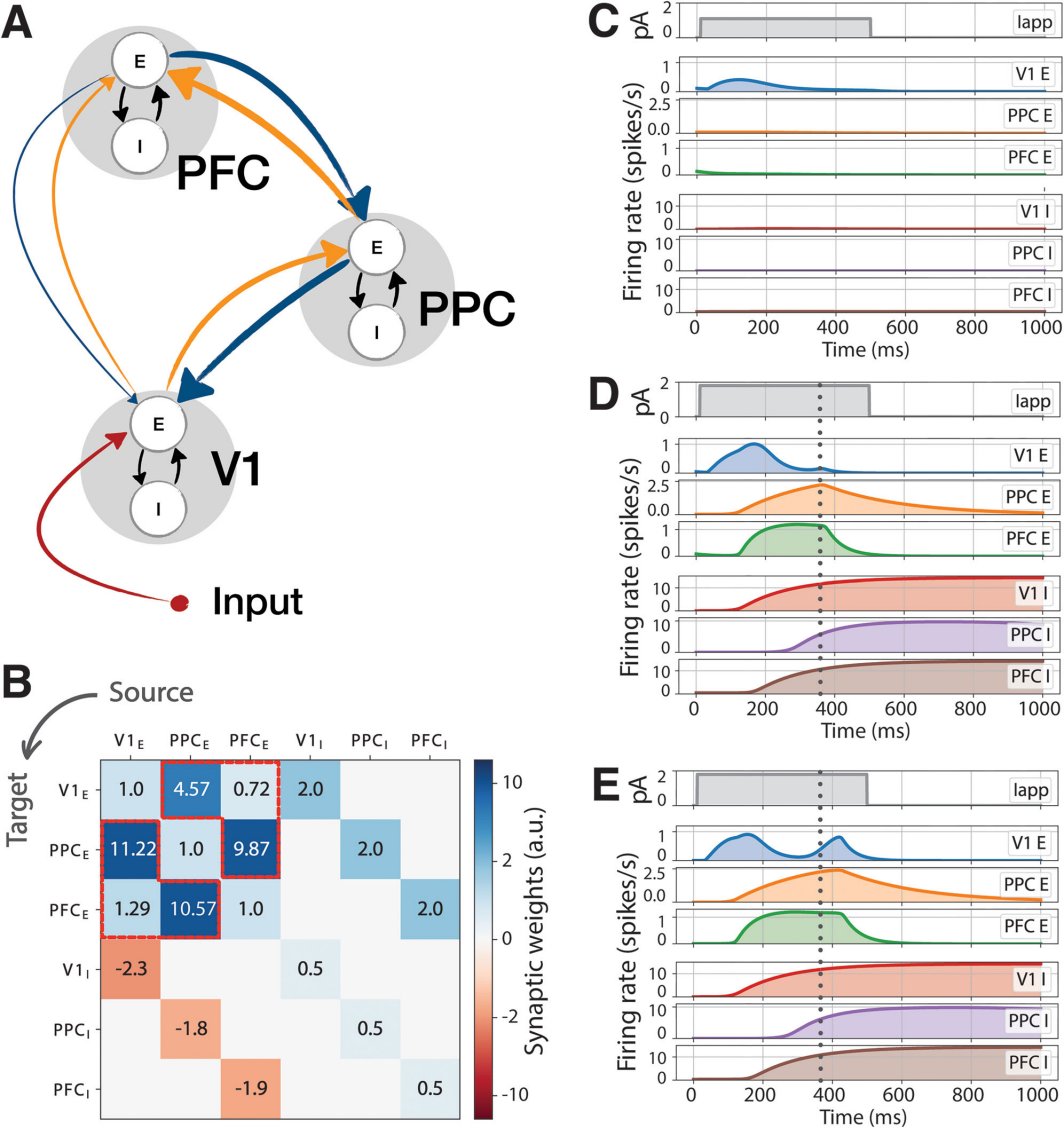
Network architecture and activity in the nominal setup. ***A***, Network schematic. We developed a minimal network model with an excitatory (E) and an inhibitory (I) node in three cortical areas: V1, PPC, and PFC. Orange (blue) arrows indicate feedforward (feedback) connections, whose values were determined based on anatomical connectivity—roughly indicated by arrow thickness; see also panel ***B***. Connections between excitatory and inhibitory nodes (black arrows) were calibrated to match experimental results. External input was applied to V1 (red arrow) to simulate visual stimuli. ***B***, Synaptic weights between nodes. The top left 3 × 3 block corresponds to nonlocal connections (matrix W, see STAR methods), while the other three blocks correspond to local couplings *γ_i_*. Values in highlighted cells (red lines) were experimentally derived. All other values were calibrated. ***C–E***, Example firing rate traces in the three regions for two different values of applied current: ***C***, low current *I*_max _= 0.8 pA; ***D***, ***E***, medium current *I*_max _= 2 pA. At medium currents a feedback bump may (***D***) or may not (***E***) appear depending on small changes in initial conditions. In ***C–E***, row 1 reflects the input, rows 2–4 the activity of excitatory nodes, and rows 5–7 the activity of inhibitory nodes.

We modeled a network of three regions, namely, V1 (primary visual cortex), PPC (posterior parietal cortex), and PFC (prefrontal cortex). Each region comprises one excitatory and one inhibitory population (Fig. 2, schematic), and the activity of each population is described by a neural mass model (Ermentrout and Cowan, 1980; Ermentrout and Terman, 2010; Bressloff, 2014; Chaudhuri et al., 2015; Joglekar et al., 2018). The model describes the evolution of the average population firing rates. Such models are macroscopic in nature; that is, they describe population activity, as opposed to single-neuron activity. Populations are connected through weighted links, which represent anatomical connectivities. Neural mass Wilson–Cowan models, such as the ones described below, are an established framework to investigate large-scale neuronal dynamics (Ermentrout and Cowan, 1980; Ermentrout and Terman, 2010; Bressloff, 2014; Chaudhuri et al., 2015; Joglekar et al., 2018).

The *i*th cortical area in the network evolves according to the following equations:

$$\begin{array}{c} \tau_{i}^{\;E}\frac{d}{dt}u_{i}^{E}=-\beta_{i}^{E}u_{i}^{E}+F_{i}^{E}\left(\gamma_{i}^{\;\mathrm{EE}}u_{i}^{E}+\gamma_{i}^{\mathrm{EI}}u_{i}^{I}+\underset{\;j\neq i}{\sum }\;W_{ij}u_{j}^{E}+I_{\mathrm{app},i}(t)\right),\\ \tau_{i}^{\;I}\frac{d}{dt}u_{i}^{I}=-\beta_{i}^{I}u_{i}^{I}+F_{i}^{I}(\gamma_{i}^{\mathrm{II}}u_{i}^{I}+\gamma_{i}^{\mathrm{IE}}u_{i}^{E}), \end{array}$$

where the superscripts E,I label excitatory and inhibitory variables, respectively. The firing rate $u_{i}$ of the *i*th population has characteristic time constants $\tau_{i}$, and it evolves according to two main contributions: a damping term proportional to $\beta_{i}$ and a nonlinear synaptic term, collecting inputs from the network. Our network is formed by three main brain regions (V1, PPC, and PFC); hence we set $u_{1}=V1$, $u_{2}=\mathrm{PPC}$, and $u_{3}=\mathrm{PFC}$, each endowed with an excitatory and inhibitory node, thereby obtaining a network with six nodes.

The local couplings are denoted by $\gamma_{i}^{\;kl}$ where k,l=E,I. Inhibitory populations are connected only locally, whereas excitatory populations have local as well as long-range connections. Long-range connections are mutual, all-to-all, and, in general, asymmetric. This means that, while each population is connected to all the others, the respective weights have different strengths. We encode the link from the excitatory population j to the excitatory i in a matrix using the equation:

$$W_{ij}=\frac{\mathrm{number}\;\mathrm{of}\;\mathrm{neuronal}\;\mathrm{projections}\;\;\mathrm{from}\;j\;\mathrm{to}\;i}{\mathrm{total}\;\mathrm{number}\;\mathrm{of}\;\mathrm{neuronal}\;\;\mathrm{projections}\;\mathrm{to}\;i}.$$

Nominal values of $W_{ij}$ (see highlighted entries in the top left 3 × 3 block of Fig. 2*B*) have been taken according to recent data on mice (Harris et al., 2019; Knox et al., 2019). This allows to develop a model with faithful connectivity between cortical regions.

The nonlinear function $F_{i}^{E}$ is sigmoidal:

$$F_{i}^{E}(I)=\frac{1}{1+e^{-\mu_{i}^{E}(I-\nu_{i}^{E})}},$$

and a similar expression holds for $F_{i}^{I}$. The parameter $\mu_{i}^{E}$influences the sharpness of the sigmoid, while $\nu_{i}^{E}$ determines the threshold at which the nonlinear firing response is triggered. Finally, we model a network receiving an external stimulus in V1; hence, $I_{\mathrm{app}}(t)$ is different from 0 only in V1, so $I_{\mathrm{app},i}=0$ for i=2,3 and it is a step function for $V_{1}$:

$$I_{\mathrm{app},1}(t)=\{\begin{array}{cc} I_{\max} & 0<t\leq T,\\ 0 & t>T. \end{array}).$$

Nominal values of parameters are reported in the next paragraph. Parameter variations are discussed in the main text.

The described equations are numerically integrated using the function ode23s in Matlab, which is based on a modified Rosenbrock formula of order 2 (Shampine and Reichelt, 1997).

#### Numerical parameter values

Connection strengths between areas (matrix W; Table 1) were taken from recent experimental data (Knox et al., 2019). All other model parameters (Tables 2–4) were manually calibrated to enable the excitatory nodes to reproduce patterns of activity comparable with those observed in vivo and reported earlier. As the model describes population activity, its variable $u_{i}$ refers to the average firing rate of single neurons within population i. As customary in mean-field models, some parameters refer to single neurons, while others to entire population. For instance, the input current $I_{\mathrm{app}}$ is interpreted as the average external current received by a single neuron (measured in pA) and similarly for coupling and synaptic constants. On the other hand, characteristic timescales refer to populations.

**Table 1.** Matrix W: connectivity between excitatory variables (units: pA/Hz)

	V1	PPC	PFC
V1		11.22	1.29
PPC	4.57		10.57
PFC	0.72	9.78	

**Table 2.** Gamma parameters (units: pA/Hz)

	V1	PCC	PFC
$\gamma^{\mathrm{EE}}$	1	1	1
$\gamma^{\mathrm{EI}}$	2.3	1.8	1.9
$\gamma^{\mathrm{IE}}$	2	2	2
$\gamma^{\mathrm{II}}$	0.5	0.5	0.5

**Table 3.** Parameters of the firing rate function

	V1	PPC	PFC
$\mu^{E}\;[1/\mathrm{pA}]$	3	2	2
$\mu^{I}\;[1/\mathrm{pA}]$	2	2	2
$\nu^{E}\;[\mathrm{pA}]$	2	4	2
$\nu^{I}\;[\mathrm{pA}]$	0.3	0.3	0.3

**Table 4.** Characteristic times and decay constants

	V1	PCC	PFC
$\tau^{\;E}\;[\mathrm{ms}]$	30	200	38
$\tau^{\;I}\;[\mathrm{ms}]$	10	10	10
$\beta^{E}$	0.8	0.9	3.8
$\beta^{I}$	0.07	0.1	0.07

#### Noise in the initial conditions

We investigated the network behavior when the nominal setup is perturbed by noise, by sampling the initial conditions from a random, uniform distribution: *u_i_*(*t* = 0) ∈ [0, *δ*].

The analysis of network behavior under noise is obtained by with 50 realizations for each network setup. We fixed *δ *=* *0.05 spikes/s, as this value was one order of magnitude smaller than the typical scales of the excitatory firing rates, and we additionally studied the effects of varying δ.

#### Instantaneous inactivation and connectivity morphing parameter

We performed two experiments to examine the robustness of the network behavior with respect to changes in the coupling between areas and also to infer which nodes are most relevant for the formation of the late activity bump. First, we introduce a connectivity morphing parameter which amplifies (*α *> 1), dampens, or suppresses (*α *< 1) one or more synaptic connections $\left(W_{ij}\right)$ with respect to their nominal value (*α *= 1), via the following transformation:

$$W_{ij}^{\mathrm{new}}=\alpha W_{ij}.$$

This transformation is performed at the initial time and allows for intermediate states of weakened connections.

In a second experiment, we consider instantaneous inactivation of certain synaptic connection: at specific time T*, we set one or more entries of the connectivity matrix $\left(W_{ij}\right)$ to zero:

$$W_{ij}(t\geq T^{*})=0.$$

### Quantification and statistical analysis

#### In vivo recordings: data analysis

All data analysis was performed in Matlab 2021b (MathWorks).

#### In vivo recordings: sensory-evoked and task-related responses

For each single neuron identified through the spike sorting procedure (V1, 594 neurons; PPC, 529 neurons; ACC, 629 neurons), we computed the average peristimulus time histogram (PSTH) aligned to the onset of visual changes, separately for hit and miss trials, as well as for small and large visual changes. PSTHs were computed with a 10 ms time bin and smoothed with a Gaussian window (standard deviation, 25 ms). Each PSTH was baseline corrected; i.e., we subtracted the average activity computed in the [−500, −10] ms window with respect to stimulus onset.

#### In vivo recordings: sensory-evoked and task-related responses—previously released dataset

The dataset used for the Steinmetz et al. (2019) study was downloaded from https://figshare.com/articles/dataset/Dataset_from_Steinmetz_et_al_2019/9598406 and analyzed using the same approach described above. Trials were pooled together based on whether a hit or miss was observed and separately for visual contrasts of 25, 50 and 100%.

#### In vivo recordings: statistical analyses

Differences between sensory-evoked responses were assessed using a permutation-based approach. For each pair of conditions to be tested (e.g., hit trials to high vs low saliency stimuli), we used the corresponding single-neuron PSTHs to compute the difference between average responses (across neurons) separately for each time bin. We then randomly swapped the trial identify of each PSTH, separately for each neuron, and computed the corresponding response difference. This was repeated 1,000 times. We then ranked, separately per time bin, the actual response difference between two conditions compared with the values obtained through random permutations. If the actual response difference was higher than 95% of the values obtained through random permutations, a difference was considered to be significant, and the corresponding *p* value was computed as the fraction of randomly obtained values which was higher than the actual difference. All *p* values were then corrected for the false discovery rate (Bonferroni’s correction). To compute if an area encoded differences between high and low saliency stimuli, we further specified that this difference had to be present for both hit and miss trials, to prevent any interaction effect. Similarly, any hit/miss difference had to be present for both low and high saliency stimuli.

#### Model: neural activity measure

We defined an integral measure which counts the cumulative number of spikes in V1, from time $t_{\mathrm{init}}$ to time $t_{\mathrm{end}}$:

$$\begin{array}{c} S=\int_{t_{\mathrm{init}}}^{t_{\mathrm{end}}}u_{1}^{E}(t)dt, \end{array}$$

where $u_{1}^{E}(t)$ is the firing rate of the excitatory population in the primary visual cortex $V_{1}$. We set $t_{\mathrm{init}}$ so as to start counting spikes after a first (stimulus-induced) bump occurred and use S to determine whether a second (feedback-induced) bump was present in V1.

#### Code accessibility

The code/software described in the paper is freely available online at https://github.com/simoneciceri/IAS_project. The code is available as Extended Data.

**Figure 3.**
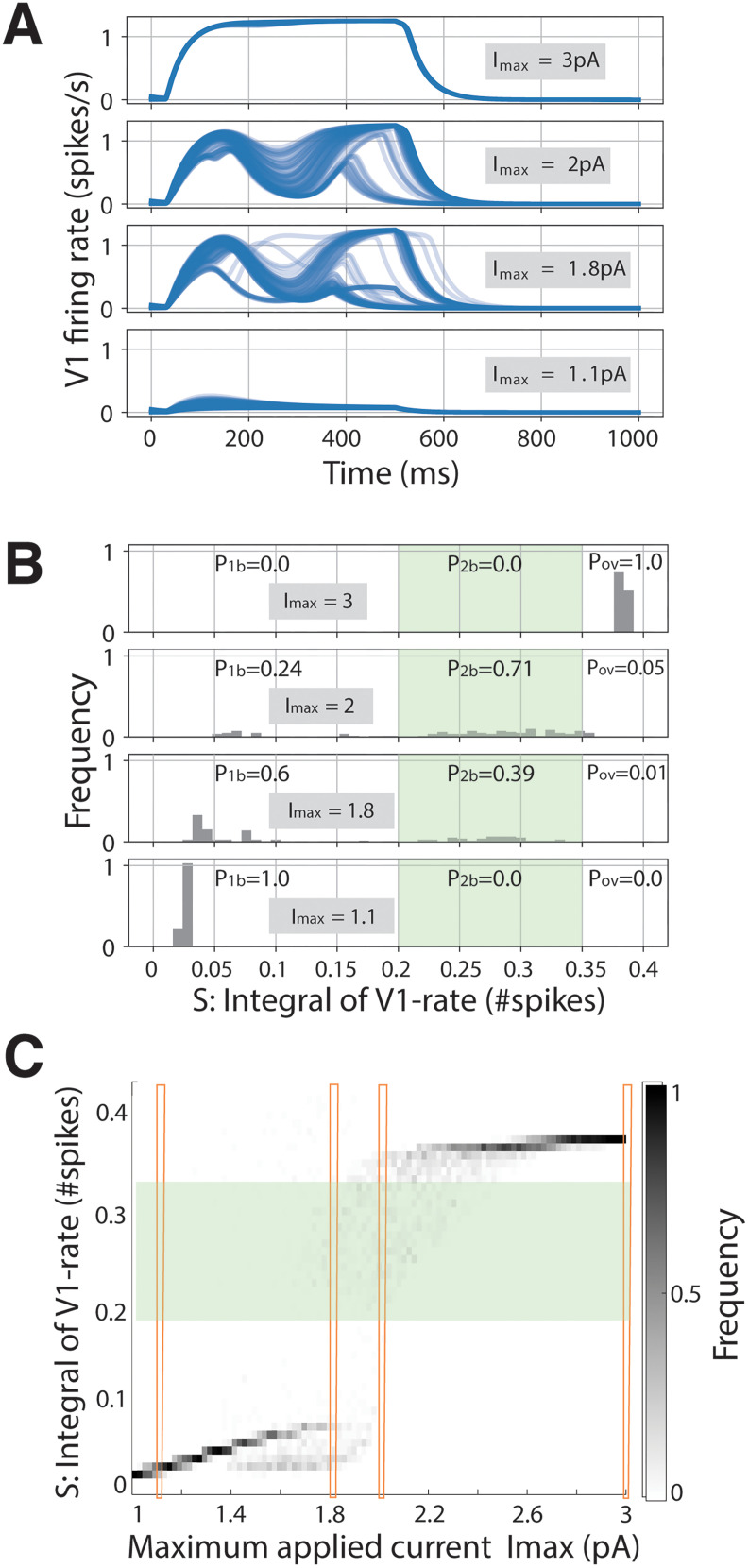
Network activity as a function of initial conditions and input currents. ***A***, Examples of activity trajectories (random initial conditions) for different input currents, in the nominal setup of parameters. ***B***, Distributions of integral quantity S for the trajectories in ***A***. A late-activity bump is detected when the integral of V1 firing rate S lies in the interval [0.2, 0.35], highlighted in green. ***C***, Frequency distribution of S for different values of applied current *I*_app_. Red columns highlight the values displayed in ***B***. The statistics is obtained over 100 different realizations for every value of *I*_max_, with initial conditions sampled from a uniform, random distribution *u_i_*(*t* = 0) ∈ [0, 0.05] spikes/s.

**Figure 4.**
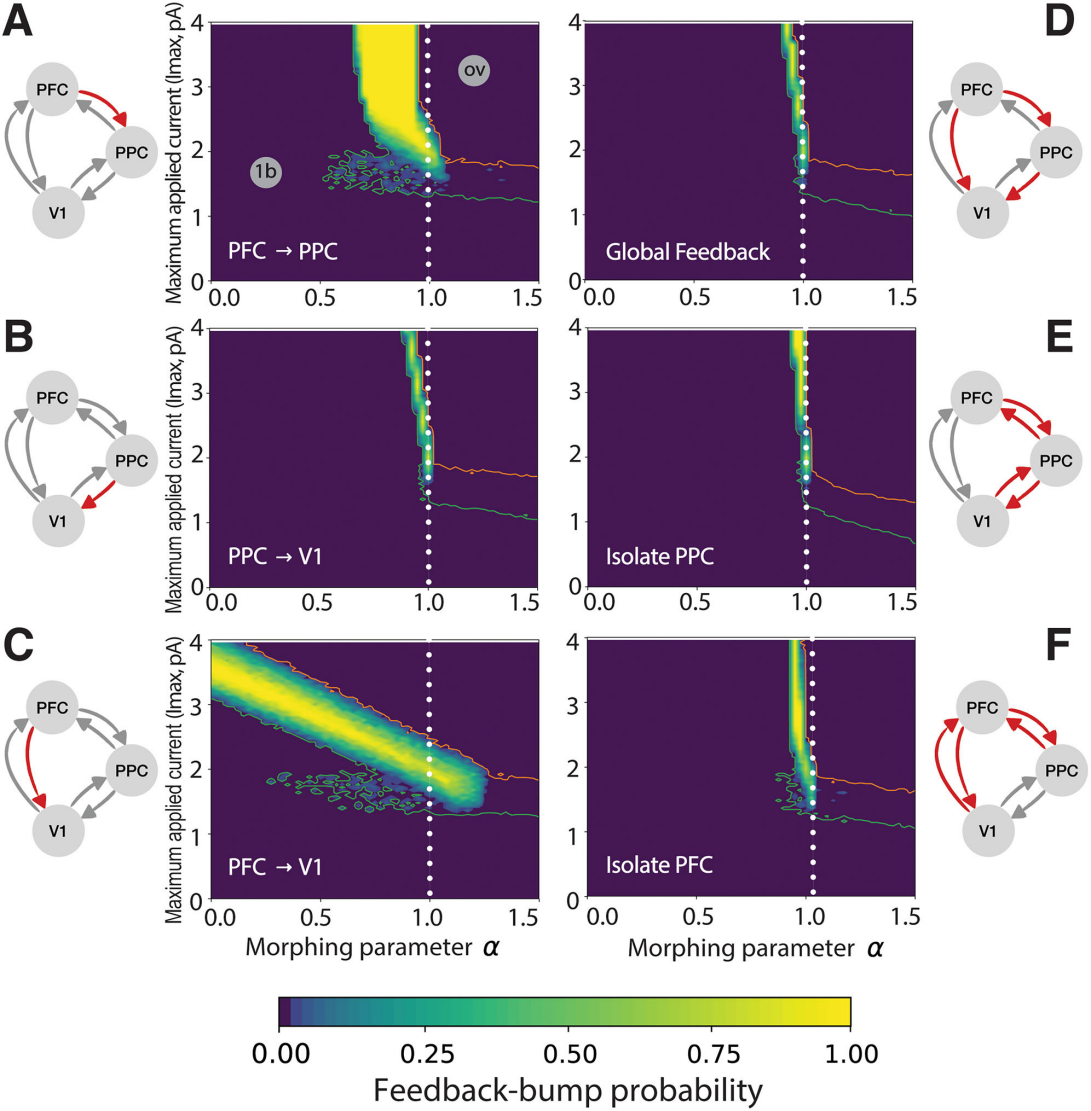
Probability of observing a late-activity bump, in the plane of parameters (*α*, *I*_max_), for the sets of connections considered. In each panel, heatmaps show the probability of observing both an early and late activity bumps *P*_2b_ as a function of applied input current *I*_max_ and morphing parameter *α*, applied to a different set of connections. The green and orange lines represent isolines of *P*_1b _= 99% and *P*_ov _= 99%, respectively, which border regions dominated by single bumps/inactivity and overshooting (see gray labels on the top-left panel). A white, dotted line marks the nominal setup (*α* = 1). For each panel, morphed connections are colored in red on the respective network scheme. For each couple of (*α*, *I*_max_) values, we considered 50 random initial conditions (see Materials and Methods).

10.1523/ENEURO.0191-24.2024.d1 Extended Data 1 The file *code.zip* includes all code used to generate the figures and perform the analyses presented in this manuscript. The folder "./ephys analysis/" contains code and instructions concerning experimental data and Figure 1. The files "Figure2CDE.m", "Figure3AB.m" and "Figure3C.m" simulate the model in the nominal setup, and generate the corresponding figures. The file "Morphing.m" allows changing the connectivity strengths with respect to the nominal setup, and plotting the resulting trajectories. The folder "./Figure4/" contains instructions, code and data concerning Figure 4. Download Extended Data 1, ZIP file.

## Results

### Detection of visual stimuli is coupled to large-scale activity patterns in the dorsal cortex

We first aimed to replicate and expand earlier reports that visual detection in mice correlates with a bimodal response pattern in V1 (Oude Lohuis et al., 2022c) and with the emergence of report-related activity across multiple cortical regions (Allen et al., 2017; Pho et al., 2018; van Vugt et al., 2018; Steinmetz et al., 2019; Yin et al., 2020; Zatka-Haas et al., 2021). To this aim, we first analyzed neuronal activity collected in head-fixed mice performing an audio-visual change detection task (Oude Lohuis et al., 2022b,c, 2024; Fig. 1*A*). Mice were trained to report the change in the orientation of the presented visual stimulus, by performing—for instance—a left lick, and a change in the pitch of the presented auditory stimulus by performing a right lick (with contingencies counterbalanced across mice; see Materials and Methods for details). In this report we only focus on the processing of the visual stimuli. Multi-area laminar probe recordings were performed in the primary visual cortex (V1), posterior parietal cortex (PPC), and anterior cingulate cortex (ACC; Fig. 1*B*). We computed stimulus-evoked spiking responses across the three areas as a function of the saliency of the visual stimulus (threshold or max change) and based on whether a stimulus was detected (hit trial) or not (miss trial). Trials from the max change condition will be referred to as “high saliency” and trials from the threshold condition as “low saliency” from here on. Of relevance, previous studies indicated that neuronal responses in both PPC and V1 did not show major deviations based on whether licking responses to full-field visual stimuli had to be done toward a detector positioned toward the left or right side of a mouse's snout (Oude Lohuis et al., 2022b,c, 2024).

In V1, we observed a bimodal pattern of activity: an early-onset wave of sensory-evoked activity, lasting until ∼200 ms after stimulus onset, followed by a late-onset wave which was mainly encoding whether a trial was a hit or miss (cf. Oude Lohuis et al., 2022c; Fig. 1*C*). Early sensory-evoked activity did not differ between hit and miss trials, but firing rates were positively correlated with the saliency of visual stimuli (Fig. 1*C*). Instead, late activity encoded both whether a trial was a hit or miss, as well as whether the sensory input was strong or weak (Fig. 1*C*; cf. Oude Lohuis et al., 2022c). Activity in PPC and ACC mainly encoded differences between hit and miss trials, although a generalized increase in firing rates could be observed as a consequence of the presentation of sensory stimuli (Fig. 1*C*; cf. Oude Lohuis et al., 2022b).

To verify that these results were not specific to our experimental protocol, we also analyzed recordings from another previously published experiment (Steinmetz et al., 2019). In this paradigm, mice had to identify which of two visual stimuli presented in the right and left hemifield had the highest contrast and rotate a wheel to move the highest-contrast sensory stimulus toward the center of the screen (Fig. 1*D*). We computed sensory-evoked responses as a function of both stimulus contrast (difference between the contrast of the two presented Gabor patches) and hit/miss responses, for trials in which the highest-contrast stimulus was shown in the hemifield of view contralateral to the recorded hemisphere. We analyzed neuronal responses in areas corresponding to those analyzed in the previous set of experiments [V1; VISa and VISam (data not shown), which are two secondary visual cortices spatially overlapping with PPC; ACC], as well as in a region broadly defined as the supplementary motor cortex (MOs), where report-related activity has been shown to originate (Allen et al., 2017; Fig. 1*B*). Results were in line with those that we observed in the first dataset. V1 showed a bimodal pattern of activity, with an early sensory-evoked response (that, however, in contrast with our dataset, did not encode stimulus saliency) followed by a report-related bump in activity (Fig. 1*E*). Activity in higher-order regions followed the early response displayed in V1 and was only report related. These results suggest that the spatiotemporal progression of visual- and report-related activity is mostly independent from the details of the task being performed. Report-related activity, however, could reflect several factors besides stimulus detection, such as motor correlates and reward expectation (Stringer et al., 2019; Avitan and Stringer, 2022; Bimbard et al., 2023; Pennartz et al., 2023; Oude Lohuis et al., 2024). To better understand what the late component of V1 activity reflects, we also plotted neuronal responses during false alarms—when a response is performed in the absence of a visual stimulus—and error trials, when animals perform an incorrect response (Extended Data Fig. 1-1). False alarms in general did not correlate with any clear neuronal activity, for what pertains the data we collected (Extended Data Fig. 1-1*A*; note that we did not observe a sufficiently high number of error trials). For the data presented in the Steinmetz et al. (2019) manuscript, both false alarms and errors correlated with an increase in firing rates in frontal and association areas (MOs, ACC, and PPC; Extended Data Fig. 1-1*B*) but strikingly did not display a clear late wave of activity in V1. Thus, nonsensory activity has been previously observed in V1 and has been shown to reflect several factors including corollary discharges related to spontaneous and task-related movement. However, for what specifically pertains the late wave of activity that emerges in hit trials, this predicts the upcoming report and might also correlate with other factors such as reward but does not purely reflect motor actions. Nevertheless, an in-depth assessment of this aspect is beyond the scope of this study.

Earlier findings (Allen et al., 2017; van Vugt et al., 2018; Yin et al., 2020) indicated that report-related activity showed an earliest peak in the prefrontal regions, followed by PPC and V1. Our results suggest a similar picture for what pertains higher-order regions, with prefrontal areas (ACC, MOs; Le Merre et al., 2021) showing earlier indications of hit/miss differences compared with PPC. The relative timing of the appearance of report-related activity in V1 is, however, less clear (compare Fig. 1*C*,*E*) but is overall very close to that observed in the prefrontal regions (Allen et al., 2017; van Vugt et al., 2018; Yin et al., 2020). Thus, while the prefrontal regions remain the most likely candidate for the origin of report-related activity—as supported by the causal experiments performed by Allen et al. (2017)—the mechanistic pathway via which this form of activity reaches other cortical areas remains unclear.

### A minimal network model reproduces the spatiotemporal propagation of visual- and report-related activity

In order to understand the possible network-level mechanisms underlying the spatiotemporal propagation of visual- and report-related activity across the cortical areas from which we analyzed neuronal activity, we developed a minimal mean-field computational model of the cortical network that: (1) uses available connectomic data (Ermentrout and Cowan, 1980; Ermentrout and Terman, 2010; Bressloff, 2014; Chaudhuri et al., 2015; Joglekar et al., 2018) and (2) is calibrated using in vivo recordings. For this reason, the model only includes the three cortical areas from which we analyzed in vivo neuronal recordings: V1, PPC, and PFC (Fig. 2*A*). The activity in each area is modeled with a firing rate neural mass model comprising one excitatory and one inhibitory population. Firing rate models of this type are a well-tested tool to describe macroscopic neuronal dynamics, as they average single-neuron spike rates (Ermentrout and Cowan, 1980; Ermentrout and Terman, 2010; Bressloff, 2014; Chaudhuri et al., 2015; Joglekar et al., 2018). Within each mass, the synaptic dynamic has a tunable dispersion time, and oscillatory dynamics are possible because of the coupling between the excitatory and inhibitory population (Coombes and Wedgwood, 2023). We also adopted a classical nonlinear sigmoidal firing rate for each neuronal population (see Materials and Methods for a complete description), which is standard for neural mass models in the literature (Ermentrout and Cowan, 1980; Ermentrout and Terman, 2010; Bressloff, 2014; Chaudhuri et al., 2015; Joglekar et al., 2018).

The mean field model comprises a total of six neuronal populations, two in each cortical area, which feature local as well as long-range connections. More precisely, the excitatory–inhibitory pair in each cortical area is fully connected and should be taken together as a model of a cortical area, where the excitatory node has been fitted to experimentally collected neuronal activity and therefore represents the output of an area (in terms of “firing” activity) and all other variables represent hidden state variables. In addition, there are long-range excitatory connections to and from each cortical area. Crucially, connection strengths between areas were taken from recent experimental data (Knox et al., 2019; Fig. 2*B*, highlighted entries). In particular, we employed values of directed connection density between V1, secondary visual areas A and AM (which are considered the mouse homologue of PPC; Driscoll et al., 2017; Pinto et al., 2019; Arlt et al., 2022; Oude Lohuis et al., 2022b), and MOs (which is considered as a component of PFC; Le Merre et al., 2021) and is thought to be the key cortical area mainly in view of generating report-related activity (Allen et al., 2017; Steinmetz et al., 2019). All other model parameters were calibrated (see Materials and Methods) to enable the excitatory nodes to reproduce patterns of activity comparable with those observed in vivo, as reported in earlier sections. During the tuning procedure all parameters in the model (characteristic rise/decay times, activation of the nonlinear firing rate functions, and local excitatory-inhibitory coupling strengths) were calibrated, while the interareal connections were kept fixed, because we had direct access to experimental data on these parameters. In this way we could test to what extent the generation and propagation of report-related activity in the three cortical areas on which we focus is shaped by corticocortical connectivity. Importantly, as our model comprises neural masses that jointly mimic the activity of whole cortical areas, all parameters except the activity of excitatory nodes do not represent measurable variables, but rather hidden state variables or input parameters that do not aim to model specific single-neuron parameters. For instance, while input currents are measured in picoampere, they reflect input to a whole cortical area and not to single neurons.

We modeled a visual stimulus via an applied transient step current, with varying intensity, on the excitatory population of V1 (Fig. 2*A*; Fig. 2*C–E*, *I*_app_ time traces), and monitored the elicited cortical firing rate response in excitatory and inhibitory populations of V1, PPC, and PFC (whose time traces are also seen in Fig. 2*C–E*). To calibrate the model, we applied a visual input lasting for 500 ms to the excitatory V1 node and replicated the following experimental results. First, when subjected to a sufficiently strong stimulus, V1 activity displayed an early-onset response peaking ∼100–200 ms (before the termination of the visual stimulus) that then dropped to lower values (Fig. 2*C*). This reproduces the adaptation to stimuli typically observed in the visual cortex (Fig. 1; Supèr et al., 2001; Steinmetz et al., 2019; Kirchberger et al., 2021; Oude Lohuis et al., 2022c). Second, high-amplitude visual stimuli evoked a bimodal V1 response, that is, an early-onset peak of activity followed by a later peak (Fig. 2*E*). This second peak was absent if the stimulus had a low-amplitude (low salience stimulus) and accounts for the report-related activity observed in vivo (Fig. 1, compare hit trials). While the model itself does not include an actuator stage to perform an actual report, we consider the emergence of the late-activity bump to represent an instance of stimulus detection by the prefrontal/premotor cortical regions. This mimics the spatiotemporal time course of sensory detection, as can be observed from the neural recordings shown in Figure 1 and from the related publications (Steinmetz et al., 2019; Oude Lohuis et al., 2022c).

Following this tuning procedure, we observed that, when the network was subjected to stimuli of various intensity for 500 ms, it displayed a strongly nonlinear response for the late peak of report-related activity: for smaller inputs $\left(I_{\max}=1.1\;\mathrm{pA}\right)$ V1 was activated, but the signal did not significantly propagate in the network and did not trigger a second, feedback-dependent bump in activity (Fig. 2*C*, excitatory nodes in row 2–4). For higher input strengths $\left(I_{\max}=2\;\mathrm{pA}\right)$, the network displayed the late activity bump in many, but not all, realizations (compare Fig. 2*D*,*E*). To run the model, we first set the initial value of the firing rate in each of the excitatory and inhibitory population; that is, we set initial conditions prior to the initiation of the stimulus. In the simulation, each population is set to a random initial state with a small variance. In particular, we found that initial conditions varying within 10^−2^ spikes/s, simulating noisy initial data, trigger or suppress the occurrence of the late activity bump.

This is in line with experimental findings showing that, when subject to a sufficiently large stimulus, large late-latency activity arises with high probability, but not with certainty (van Vugt et al., 2018). The probabilistic nature of this response is analyzed in detail in later sections. Before addressing this aspect, we observed the dynamics in each neuronal population and noted that the occurrence of a late-latency activity bump appears to be feedback-induced. The external stimulus activated V1 which, in turn, following a feedforward chain, activated PPC and PFC. The activity in the latter areas reached a peak (Fig. 2*D*,*E*, vertical time marker) before decaying owing to local (intrapopulation) inhibition. The time marker aligned remarkably well with the small late-latency activity in V1, signaling the onset of a feedback mechanism (from PFC and PPC back to V1; Fig. 2*A*, schematic). In realizations in which the late activity bump occurred, it was again PFC and PPC that displayed a peak preceding the late, report-related activity in V1, in line with a feedback mechanism. This claim will be further substantiated in the following sections.

Thus the model, using a set of nominal parameters, was able to qualitatively reproduce the types of activity we observed in vivo. While the model was specifically tuned to reproduce V1 activity, we also obtained comparable patterns of activity in PPC and PFC, indicating that the model could be used to study the mechanisms underlying the propagation of activity across cortical areas during sensory motor transformations. In particular, we focused on studying the role of feedback connections in the genesis of the late activity bump, i.e., of report-related activity.

We highlight that the time courses of the activity of excitatory nodes are strongly determined by the inhibitory ones: in Figure 2*D*,*E*, it is visible that inhibitory nodes in each cortical area activate after the corresponding excitatory node, and this determines the rise-and-fall behavior in the latter. It is known that neurotransmitter release in excitatory and inhibitory populations are affected by timescale separation between signals (Rodrigues et al., 2016). Our model achieves the delayed inhibitory activation using timescale separation between excitatory and inhibitory rising times (see discrepancies in the parameters $\tau^{\;E}$ and $\tau^{\;I}$ in Materials and Methods). However, we also must point out that, for the purposes of this study, we did not calibrate the dynamics of inhibitory nodes to match experimental results, but only tuned their parameters so that excitatory nodes would show realistic behaviors. For this reason, we will only focus on excitatory nodes in the rest of the manuscript.

### The likelihood of late activity bumps is influenced by variations in internal state

As we have seen above, when the network is in the nominal setup and the visual stimulus is sufficiently high, a late activity bump occurs with a given probability, upon perturbing the initial state of the system. We investigated systematically this scenario by running 100 simulations during which the initial state of the excitatory V1 population was picked randomly and uniformly between 0 and 0.05 spikes/s, thereby imposing a small variance in the internal state (Fig. 3*A*) that is in line with experimental observations linking cortical state fluctuations to perception (Supèr et al., 2003; McGinley et al., 2015a,b; Speed et al., 2019; Samaha et al., 2020). We observed that early V1 responses elicited by either small $\left(I_{\max}=1\;\mathrm{pA}\right)$ or large $\left(I_{\max}=3\;\mathrm{pA}\right)$ input currents were not affected by such small variations, as trajectories were grouped together as simulation time progressed. On the other hand, intermediate currents $\left(I_{\max}=1.8-2\;\mathrm{pA}\right)$ considerably propagated the initial uncertainty: a late activity bump occurred often, but the fine details of the trajectory could differ. These findings further support the conclusion that the network in the nominal setup supports robustly self-generated late-latency activity bumps. However, more delicate questions arise: given a fixed set of network parameters, how often does the network generate such a bump? And further: how do changes in the network parameters affect this likelihood? To address these questions, we developed first a mathematical index to track late-latency V1 activity.

We therefore introduced a cumulative (integral) spiking measure *S*, with the view of determining the likelihood of late-latency activity in V1. For each of the V1 traces seen in Figure 3*A*, we counted the average cumulative number of spikes occurring after the early activity bump in V1 (Materials and Methods). More precisely, we disregarded the trajectory before the reference time $t_{\mathrm{init}}$ = 250 ms, because this is the characteristic time in which the early activity bump occurs (Del Cul et al., 2007; Oude Lohuis et al., 2022c), and then we calculated the area under the curve (proportional to the average number of population spikes) between $t_{\mathrm{init}}$ and the end of the simulation, $t_{\mathrm{end}}=1,000\;\mathrm{ms}$, during which the late activity bump may occur. We expected trajectories with $I_{\max}=1.1\;\mathrm{pA}$ (Fig. 3*A*) to have a very small cumulative spike number *S*, because they did not display a late activity bump. Indeed, the histogram in Figure 3*B* with $I_{\max}=1.1\;\mathrm{pA}$ shows that, in all such trajectories, fewer than 0.05 spikes were observed in V1, on average, after the early activity bump, in the time interval [250, 1,000 ms]. On the contrary, a fully saturated response, in which firing rate reach the maximum value allowed by the model's equations (Fig. 3*A*; $I_{\max}=3\;\mathrm{pA}$), is characterized by a large *S*, and indeed the histogram in Figure 3*B* (with $I_{\max}=3\;\mathrm{pA}$) shows that all such trajectories had >0.35 spikes after the first bump, on average. Finally, a late activity bump was characterized by an intermediate value of *S*: with $I_{\max}=1.8-2\;\mathrm{pA}$ we observed a clear separation in the histogram of *S*. Therefore, we can use the value of *S* to define, empirically, the occurrence of a late activity bump (Fig. 3*B*). We thus classified a V1 activity trace by the corresponding value of *S*: we labeled traces with 0<S<0.2, as displaying only the early activity bump, traces with 0.2≤S≤0.35 (Fig. 3*B*,*C*, green band) as displaying both the early and late activity bumps and those with S>0.35 as displaying an overshoot (Fig. 3*A*).

Based on the fraction of trajectories whose *S* value falls in each of these three bands, we estimated the probability of having only the early activity bump as *P*_1b_, both the early and late activity bumps as *P*_2b_ and overshoot as *P*_ov_. For example, from the histograms in Figure 3*B* with stimulus $I_{\mathrm{app}}=2\;\mathrm{pA}$, we estimated that the nominal network displays both an early and late activity bump with probability $P_{2b}=71\text{\%}$, an overshoot with probability $P_{\mathrm{ov}}=5\text{\%}$, and an early activity bump only or inactivity with probability $P_{1b}=24\text{\%}$.

### Late-latency activity relies on network feedback

Armed with a quantitative index to inspect the likelihood of late-latency activity, we investigated how this likelihood changes upon variations in the network topology. As we shall see below, this analysis revealed that the late-latency activity is feedback induced. We performed two different experiments which transform the connectivity matrix. In the first one, we perturbed the connectivity matrix at the initial time and kept the matrix constant thereafter. In the second experiment, we dynamically perturbed the matrix to explore the effects of abrupt changes to the topology of the network.

With the view of imposing changes in the network connectivity, we introduced a morphing parameter α (see Materials and Methods). When α=1, the network is in its nominal state (the one studied so far); when α>1, selected network links are strengthened; when α<1, those links are weakened; finally, when α=0, the links are absent. The name morphing parameter suggests that with this index, we can continuously transform the nominal network to intensify weaken or even suppress certain links. Therefore, we introduced a tool to causally study to what extent the specific strength of an interarea connection enables the emergence of a regime in which a sensory input to V1 can (with a certain probability) determine the occurrence of a late, report-related bump in activity.

We first used the morphing parameter to vary the strength of selected networks links from the starting time onward, beginning with the feedback link from PFC to PPC (Fig. 4*A*), signposted with a red arrow in the network schematic (mathematically, the PFC→PPC connection was scaled by factor α). We repeated the experiment of Figure 3*A*,*B* for various values of the morphing parameter ( α between 0 and 1.5) and recorded the probability of a single early bump $P1_{b}$, both early and late bumps $P_{2b}$, and overshoot $P_{\mathrm{ov}}$. We used $P_{2b}$ to derive the heatmap showed in Figure 4*A*: the lighter colors correspond to a higher probability of a late activity bump, while darker colors denote lower probability thereof. We also used isolines to indicate where the probability of a single bump $P_{1b}$ crosses 99% (green isoline) and where the probability of overshoot $P_{\mathrm{ov}}$ crosses 99% (orange isoline).

The overall information we gathered from the heatmap in Figure 4*A* can be summarized as follows. First, the network produced a late activity bump robustly with respect to changes in the PFC→PPC feedback link: light yellow areas (late-bump probabilities close to 100%) were found in a variety of network configurations (for various values of α). Second, there are regimes, labeled “ov” and “1b,” where the feedback bump was absent, but either an overshoot (ov) or an early bump only (1b) were found with probability >99%, respectively. Third, the network could produce a late activity bump even when the feedback link PFC→PPC was weakened with respect to the nominal condition, provided that the strength of the impinging stimulus was increased; this can be deduced from the yellow area in Figure 4*A* “curving upwards” toward higher values of $I_{\max}$. Finally, the feedback pathway PFC→PPC was an important player in triggering late-latency activity. While the network could compensate for the weakening of this link with a higher input to produce a late-latency bump, network configurations in which that link was either too weak or too strong failed to produce a late activity bump.

A markedly different behavior was observed when we perturbed the feedback link PPC→V1 (Fig. 4*B*). From Figure 4*B*, it can be seen that the likelihood of a late activity bump was strongly affected by changes in this feedback link. Small deviations from the nominal value of the link caused the late activity bump to disappear quickly. While the network could tolerate a weaker PFC→PPC link (Fig. 4*A*), even the slightest weakening of the PPC→V1 link caused a complete suppression of the feedback bump. This data revealed that the experimentally derived anatomical connectivity used in the nominal conditions (α=1) was crucial to obtain a late activity bump.

On the contrary, the network's activity was only minimally affected by changes to the feedback link PFC→V1. Figure 4*C* shows that feedback bumps could be produced with high probability even when this link was absent (α=0), in case the strength of the input was increased (compensating for the reduced PFC→V1 link).

For the experiments in Figure 4*A–C*, we perturbed one link at a time, but the morphing parameter can also be varied on multiple links simultaneously. In Figure 4*D*, for instance, we strengthened or weakened all the feedback pathways at once. These manipulations showed that late-latency bumps cannot exist without (or with too much) feedback. Therefore, it is the interplay between the various feedback pathways that generates the late-latency bump. This was further confirmed by the results displayed in Figure 4*E*,*F*, showing that a network in which the PFC or PPC nodes were progressively isolated (transforming the architecture into a two-node network) did not display robust late activity bumps.

**Figure 5.**
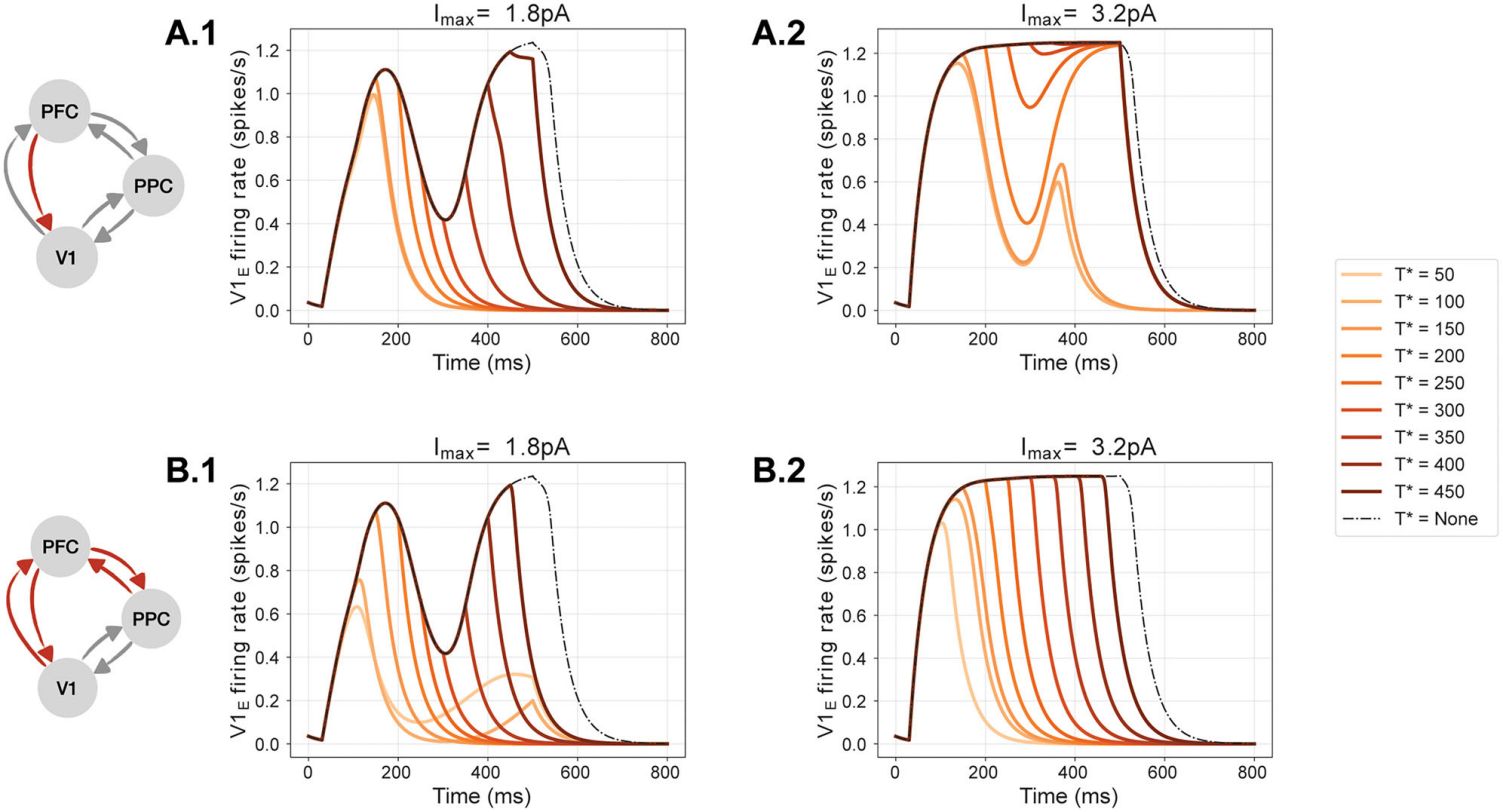
Effects of instantaneous link inactivation on feedback bumps. ***A***, We selected initial states and nominal conditions leading to late-latency activity (dashed line, T* = none). We ran this network up to a chosen time T* and then instantaneously set the PFC→V1 link to zero. For *I*_max _= 1.8 pA (***A.1***) the instantaneous inactivation prevented (T* = 50–300 ms) or suppressed (T* > 300 ms) the feedback bump in V1. Conversely, when a higher value of *I*_max_ is used (***A.2***, *I*_max _= 3.0 pA), feedback activity can be restored in V1 despite the inactivation of the PFC→V1 link. ***B.1–2***, Same as ***A.1–2*** but for the isolation of PFC from the network. Note how the feedback bump is always prevented or suppressed irrespective of the value of *I*_max_ (compare Fig. 4). Similar results were obtained for all other set of connections (compare Fig. 4).

The previous experiment modulated the strength of feedback connections over the whole simulation period. However, we expect that, if the network receives a shock in the form of instantaneous removal of certain links during sensory processing (rather than from the initial time), this will also have an impact on late-latency activity. We investigated this scenario in a further experiment: we selected initial states which, in nominal conditions, would lead to late-latency activity; we then ran this network up to a chosen time T*, at which we instantaneously set selected links to zero (see Materials and Methods). In Figure 5*A.1*, the PFC→V1 link is removed for different values of T* in the range 50–450 ms, when applying a value of *I*_max _= 1.8 pA. We found that inactivating the link at any time resulted in a quick decay in V1 activity, preventing the feedback bump to occur. In Figure 5*B*.*1*, analogous results are found when PFC is instantaneously isolated from the rest of the network. We found similar results when we removed network links from the initial time (Fig. 4).

However, as we discussed earlier, the amplitude of the external stimulus to V1 (i.e., *I*_max_) influences the genesis of the feedback bump. We thus repeated the previous experiments using a larger value of *I*_max _= 3 pA in order to establish whether sufficiently large inputs can compensate for instantaneous inactivation of the network links. In all cases, except for the PFC→V1 link, we found that large stimuli cannot overcome instantaneous inactivation of a set of links, hence impairing the generation of a feedback bump. In Figure 5*B*.*2*, representative of most cases, it can be seen that a second bump is not formed, irrespective of the value T*. On the contrary, a feedback bump is still visible when inactivating the PFC→V1 link with a T* between 150 and 300 ms, showing that feedback activity can be restored by sufficiently large external drive in spite of the inactivation of this specific link.

In summary, the analyses performed using the morphing parameter shed light on the fact that the late activity bump is dependent on feedback connections. Upon external stimulation, V1 is activated and a feedforward pathway excites PPC and PFC. From these two areas, a late-latency activity bump appears in V1, mainly owing to an indirect pathway from PFC to PPC and then to V1. It turns out that direct feedback from PFC to V1 is instead not crucial for feedback bumps to be observed in V1, because the impact of reducing this link can be easily compensated by increasing stimulus strength.

## Discussion

In this study we set out to elucidate how report-related late activity patterns observed in the cortex may mechanistically emerge as a function of the anatomically constrained connectivity between sensory, association, and prefrontal cortices. We presented evidence of two crucial stages in the processing of visual information during perceptual decision-making in mice, replicating and extending previous work in both humans and animals (Del Cul et al., 2007; Dehaene and Changeux, 2011; Allen et al., 2017; van Vugt et al., 2018; Steinmetz et al., 2019; Oude Lohuis et al., 2022c; Alilović et al., 2023). Neural recordings collected from two independent labs using two different tasks revealed that V1 firing rate at 100 ms after stimulus change was uniquely modulated by the saliency of the stimulus and not by the decision made by the mouse (Oude Lohuis et al., 2022c). In contrast, a second, later wave of V1 activity reflected a combination of stimulus saliency and decision outcome. This late wave of activity was stronger for hits than for misses and coincided with increased report-related activity in both posterior parietal and frontal cortex. Previous studies have shown that this late wave of activity is—in sensory cortices—orthogonal to sensory-evoked, early activity (Zatka-Haas et al., 2021). Moreover, the onset of this late component determines for how long V1 is necessary to detect visual stimuli: inactivating V1 after its onset does not impair detection (Oude Lohuis et al., 2002c). Some hypotheses have been made about a possible involvement in learning processes or in performing self-motion correction of visual information (Avitan and Stringer, 2022; Pennartz et al., 2023), but the function of the large increase in V1 spiking activity that predicts the upcoming detection of a visual stimulus remains poorly understood.

Here we set out to model the interactions between three key cortical regions that are thought to underlie the origin and propagation of report-related activity, to understand the mechanisms giving rise to the observed neural dynamics. Crucially, while designing the model, connection strengths between V1, PPC, and PFC were taken from recently revealed anatomical connectivity profiles between these regions. Thus, connectivity between cortical regions was imposed as a fixed constraint onto neural activity. Previous studies on the origin of report-related activity either incorporated a limited set of cortical areas without constraining interarea connectivity to realistic values (Del Cul et al., 2007; van Vugt et al., 2018) or included many cortical regions but only globally varied interareal coupling strength (Dehaene et al., 2003; Dehaene and Changeux, 2005; Castro et al., 2020). Our approach allowed to mechanistically test the role of all combinations of single and multiple feedback connections with realistic strengths, something not feasible in whole-brain models. Importantly, thanks to the close link between experiments and our model, the time-specific inactivation experiment also provides predictions that, in contrast with more complex modeling approaches, are directly testable. Specifically, by optogenetically inactivating (i.e., isolating) PPC or PFC starting from different time points following stimulus onset [see Resulaj et al. (2018); Kirchberger et al. (2021) and Oude Lohuis et al. (2022c) for a similar approach], one could test the model validity in an existing experimental setup. Nevertheless, we cannot exclude that including additional areas known to play a role in top-down modulation of sensory areas (Wimmer et al., 2015; Zhang et al., 2016; Halassa and Kastner, 2017) might have partially modified the results we obtained. However, the fact that model behavior echoed the neural data observed in vivo indicates that the model's architecture and the free model parameters were enabling physiologically plausible sensory-evoked activity for a given, anatomically based interareal connectivity matrix. Nevertheless, it is important to briefly discuss the possible role of different areas. First, the anterior cingulate cortex (ACC) sends stronger feedback projections to V1 compared with other subdivisions of PFC such as MOs (Zhang et al., 2016; Le Merre et al., 2021). However, even though ACC has been shown to significantly modulate V1 activity (Zhang et al., 2014; Fiser et al., 2016), report-related activity has consistently been found to first appear in other PFC subdivisions compatible with MOs (Allen et al., 2017; Steinmetz et al., 2019; Yin et al., 2020; Takahashi et al., 2021; Inagaki et al., 2022). Second, while we focused on PPC, other temporal association areas have been implicated in visual perception (Conway, 2018). However, at least in rodents, medial association areas such as PPC, which have been hypothesized as the rodent homolog of the dorsal stream (Wang et al., 2012; Glickfeld et al., 2014), are more strongly connected to prefrontal areas compared with temporal association cortices (Harris et al., 2019; Knox et al., 2019). Furthermore, to what extent individual areas in the mouse association cortex provide unique contributions to sensory processing or form instead a redundant network is a matter of active debate (Glickfeld et al., 2014; Jin and Glickfeld, 2020; Keller et al., 2020; Kirchberger et al., 2021; Oude Lohuis et al., 2022b,c). Therefore, we believe that considering PPC as a single network node is an important first step to better understand the role of the parietal and temporal cortex in the generation of report-related activity, to be followed up by a more detailed characterization. Finally, although we cannot exclude a role of thalamic nuclei in mediating long-range top-down modulation, the thalamus seems to mainly play a modulatory role and not directly be involved in information transfer (Wimmer et al., 2015; Halassa and Kastner, 2017).

There were at least three notable observations in our model behavior. First, increasing the input strength to V1 led to both stronger early activity waves (∼100 ms) as well as an increased likelihood of a second late wave of activity (∼400 ms), as observed in our data and in previous studies (Supèr et al., 2001; Del Cul et al., 2007). Stronger V1 input also directly led to increased late activity in PPC and PFC, temporally preceding the late wave of V1.

Second, the initial prestimulus condition of each neural node strongly determined the likelihood of the late wave to occur. In fact, we observed a regime of model parameters in which there was a clear nonlinear threshold for “igniting” this late wave, in line with the Global Workspace model (Dehaene et al., 2003; Dehaene and Changeux, 2005, 2011; Mashour et al., 2020). This is in agreement with recent observations that variations in behavioral and cortical state, associated with ongoing fluctuations in prestimulus neural activity, partly driven by neuromodulatory sources, strongly determine the likelihood that a stimulus will be perceived and reported and that late report-related neural activity in cortex is observed (McGinley et al., 2015a,b; Speed et al., 2019; Waschke et al., 2019; McCormick et al., 2020; Nuiten et al., 2023; Beerendonk et al., 2024).

Third, not only did we show that late activity in V1 was driven by feedback from higher-order regions but also, more specifically, that although PFC was necessary to generate report-related activity in V1, it exerted its final influence on V1 likely only indirectly, through PPC. While PFC activity was necessary for the buildup and initiation of the late, report-related bump in V1 activity, it was PPC that seemed to have the final veto, determining its characteristics. Removing the feedback connection from PFC to V1 had very limited influence, and the occurrence of a late wave could still be observed when input strength was increased. Thus, an interplay between frontal and parietal regions is required, and most efficient, for eliciting late feedback activity in V1. This observation is important because, in the Global Neuronal Workspace model, the frontal cortex has always been considered the site responsible for igniting the network and hence threshold setting (Dehaene, 2014; but see Sergent et al., 2021). It has been hypothesized that once the threshold for conscious report in PFC is crossed, PFC sends information to other brain regions, including the parietal cortex. We provide tentative evidence for a possible different division of labor between the frontal and parietal regions, in which the frontal cortex acts as a fast accumulator of evidence and parietal cortex as a much slower one (Kim and Shadlen, 1999; Shadlen and Newsome, 2001; but see Pinto et al., 2022). In this scenario, PFC starts to quickly feed stimulus evidence to the parietal cortex, but activity in PFC quickly reaches a ceiling level. When activity in PFC reaches such a level, this is not per se sufficient to elicit report-related activity. In contrast, the parietal cortex keeps on accumulating evidence from both the sensory and prefrontal regions over time and once PPC activity crosses a certain threshold, this triggers report-related feedback in V1. This result is a direct consequence of the known anatomically constrained connectivity between areas (Harris et al., 2019; Knox et al., 2019) and specifically from the fact that PPC has a much stronger feedback connection to V1 compared with PFC. Thereby, our study provides a novel mechanistic hypothesis on the cortical pathway via which report-related activity, a hallmark of conscious access, is generated in the frontoparietal cortex and then reaches sensory areas. Future experimental work will be required to validate our results in vivo.

## Synthesis

Reviewing Editor: Mark Laubach, American University Decisions are customarily a result of the Reviewing Editor and the peer reviewers coming together and discussing their recommendations until a consensus is reached. When revisions are invited, a fact-based synthesis statement explaining their decision and outlining what is needed to prepare a revision will be listed below. The following reviewer(s) agreed to reveal their identity: Stefano Panzeri.

Thank you for submitting your work to eNeuro!

Your paper was reviewed favorably and some minor revisions are needed. They are from one of the reviewers. Please revise your manuscript accordingly and the revisions will be reviewed again by that reviewer and me.

Reviewer 2

This manuscript uses a modeling approach to better understand a late bump of task related activity in V1 that is thought to be related to stimulus report. Specifically, the authors use their network model to dissect the contribution of anatomical pathways between V1, PFC, and PPC. They found that PPC plays an important role in facilitating late wave activity. Overall, the conclusions drawn are supported by modeling results. The manuscript and figures convey the results clearly. I have a few clarifying questions about the analyses.

1) The authors interpret the second wave of activity after stimulus onset as the late wave of activation related to "stimulus report or detection". However, other interpretations are possible which should be explored with additional analyses. For example, this activity coincides with and hence may reflect task-related and other co-occurring motor actions. Indeed, in the Steinmetz 2019 dataset, activity in the MOs and ACC peaks around the time of wheel turning. The reduction in late wave activity in miss trials is difficult to interpret since by definition there were no task-related actions in these trials. Expanding the analysis to include incorrect trials could shed more light on this issue.

2) More conceptually, what functional role does the late wave of activity play in task execution, since it occurs after the presumptive time of stimulus detection and task-related movements. Does it influence behavior at longer time scales that may manifest as trial history effects?

3) It would be useful to show task timelines in Fig. 1 so readers can better interpret the activity shown.

4) Feedforward projections target both excitatory and inhibitory neurons. Are the results qualitatively similar if inhibitory feedforward connections are added?

## Author Response

Synthesis of Reviews:

Your paper was reviewed favorably and some minor revisions are needed. They are from one of the reviewers. Please revise your manuscript accordingly and the revisions will be reviewed again by that reviewer and me.

We thank the editor and the reviewers for their careful and positive assessment of our manuscript. We have now addressed all the issues raised by reviewer 2.

Reviewer 2 This manuscript uses a modeling approach to better understand a late bump of task related activity in V1 that is thought to be related to stimulus report. Specifically, the authors use their network model to dissect the contribution of anatomical pathways between V1, PFC, and PPC. They found that PPC plays an important role in facilitating late wave activity.

Overall, the conclusions drawn are supported by modeling results. The manuscript and figures convey the results clearly. I have a few clarifying questions about the analyses.

1) The authors interpret the second wave of activity after stimulus onset as the late wave of activation related to "stimulus report or detection". However, other interpretations are possible which should be explored with additional analyses. For example, this activity coincides with and hence may reflect task-related and other co-occurring motor actions.

Indeed, in the Steinmetz 2019 dataset, activity in the MOs and ACC peaks around the time of wheel turning. The reduction in late wave activity in miss trials is difficult to interpret since by definition there were no task-related actions in these trials. Expanding the analysis to include incorrect trials could shed more light on this issue.

The reviewer is correct in pointing out that the late wave of activity almost certainly reflects more than one factor. Indeed, previous studies showed that motor activity is reflected in V1 (Avitan and Stringer, 2022; Bimbard et al., 2023; Oude Lohuis et al., 2024; Pennartz et al., 2023; Stringer et al., 2019). To enable a better understanding of the information encoded in late activity, we have now made a supplementary figure (Extended Data Figure 1-1), where we display information related to two types of incorrect trials: false alarms and errors. For what pertains the dataset we collected, however, error trials represent a small minority of all trials (in our paradigm mice tend to either miss a stimulus or respond correctly), and we therefore only present false alarms. The new plots are presented as extended data because including additional line plots in Figure 1 would have cluttered it and would have made it more difficult to read. In false alarms, mice performed a "visual" response (either a licking action or a wheel motion, based on the paradigm). In the absence of a visual stimulus. Thus, false alarms allow to see whether the late wave of activity encodes factors such as report and movement in the absence of stimulus and reward. Errors, instead, correspond to instances in which mice perform a wrong response (i.e. directed towards the wrong side, either in terms of wrong lick spout or direction of wheel motion). Errors thus allow to determine to what extent the late wave encodes the specific motor action and (the absence of) reward. False alarms in general did not correlate any clear activity, for what pertains the data we collected (Fig. 1-1A). For the data included in the Steinmetz et al. (2019) manuscript, both false alarms and errors correlated with an increase in firing rates in frontal and association areas (MOs, ACC and PPC, Fig. 1-1B), but strikingly did not display and clear late wave of activity in V1. Thus, while non-sensory activity has been observed in V1 and reflects several factors such as motor activity, for what specifically pertains the late wave of activity that emerges following a to-be-detected sensory stimulus, this predicts the upcoming reports and might reflect reward, but does not purely reflect motor responses. This is now reflected in a new paragraph in the Results section: "Report-related activity, however, could reflect several factors besides stimulus detection, such as motor correlates and reward expectation (Stringer et al., 2019; Avitan and Stringer, 2022; Bimbard et al., 2023; Pennartz et al., 2023; Oude Lohuis et al., 2024). To better understand what the late component of V1 activity reflects, we also plotted neuronal responses during false alarms - when a response is performed in the absence of a visual stimulus - and error trials - when animals perform an incorrect response (Figure 1-1). False alarms in general did not correlate any clear activity, for what pertains the data we collected (Fig. 1-1A, note that we did not observe a sufficiently high number of error trials). For the data presented in the Steinmetz et al. (2019) manuscript, both false alarms and errors correlated with an increase in firing rates in frontal and association areas (MOs, ACC and PPC, see Fig. 1-1B), but strikingly did not display and clear late wave of activity in V1. Thus, while non-sensory activity has been observed in V1 and reflects several factors such as motor activity, for what specifically pertains the late wave of activity that emerges following a to-be-detected sensory stimulus, this predicts the upcoming reports and might reflect reward, but does not purely reflect motor responses. However, an in-depth assessment of this aspect is beyond the scope of this study." 2) More conceptually, what functional role does the late wave of activity play in task execution, since it occurs after the presumptive time of stimulus detection and task-related movements. Does it influence behavior at longer time scales that may manifest as trial history effects? This is a fascinating question, to which unfortunately there is to our knowledge still no answer. Previous studies have shown that this late wave of activity is - in sensory cortices - orthogonal to sensory-evoked, early activity (Zatka-Haas et al., 2021). A recent study from our lab has shown that the onset of this late component determines for how long V1 is necessary to detect visual stimuli: inactivating V1 after its onset does not impair detection (Oude Lohuis et al., 2022). Some hypotheses have been made about a possible involvement in learning processes or in performing self-motion correction of visual information (Avitan and Stringer, 2022; Pennartz et al., 2023). We are ourselves actively working in this field, but no definitive answer is available. To address this issue, we have added the following text to the Discussion section: "Previous studies have shown that this late wave of activity is - in sensory cortices - orthogonal to sensory-evoked, early activity (Zatka-Haas et al., 2021).Moreover, the onset of this late component determines for how long V1 is necessary to detect visual stimuli: inactivating V1 after its onset does not impair detection (Oude Lohuis et al., 2022). Some hypotheses have been made about a possible involvement in learning processes or in performing self-motion correction of visual information (Avitan and Stringer, 2022; Pennartz et al., 2023), but the function of the large increase in V1 spiking activity that predicts the upcoming detection of a visual stimulus remains poorly understood." 3) It would be useful to show task timelines in Fig. 1 so readers can better interpret the activity shown.

We have expanded panels A and D in Fig. 1 to describe the timelines of the two tasks.

4) Feedforward projections target both excitatory and inhibitory neurons. Are the results qualitatively similar if inhibitory feedforward connections are added? The connection matrix of our model has been taken from available dataset for long-range anatomical connections in the mouse neocortex, and the prevalent working hypothesis in literature is that such long-range connections are mainly excitatory, although - to our knowledge - limited data is available about to what extent excitatory and inhibitory projections are targeted. Moreover, we decided to develop a model allowing us to fit as few parameters as possible throughout the paper, and therefore we have refrained from creating long-range structural inhibitory connections between different modules (excitatory and inhibitoy), as these provide wiggle room for creating or reproducing a large variety of spurious dynamics. An important point to note, furthermore, is that our model, as is, does account for feedback and feedforward functional connections engaging inhibitory modules.

However, these occurt indirectly: any inhibitory mass influences the excitatory mass locally, and hence regulates the inhibition non locally, through the structural connections. Without this mechanism, and the setup of appropriate excitatory and inhibitory time scales in each mass, we believe that it would be not possible to properly calibrate the model so as to reproduce experimental activity. For this reason, including feedforward connections targeting inhibitory neurons would not necessarily add biological plausibility to the model, and would actually disrupt its dynamics.
