# Supplementary figures and images for: The Neural and Computational Architecture of Feedback Dynamics in Mouse Cortex during Stimulus Report
Source: eNeuro. 2024 Sep 24;11(9):ENEURO.0191-24.2024. doi: 10.1523/ENEURO.0191-24.2024 (PMC11444237; doi:10.1523/ENEURO.0191-24.2024)

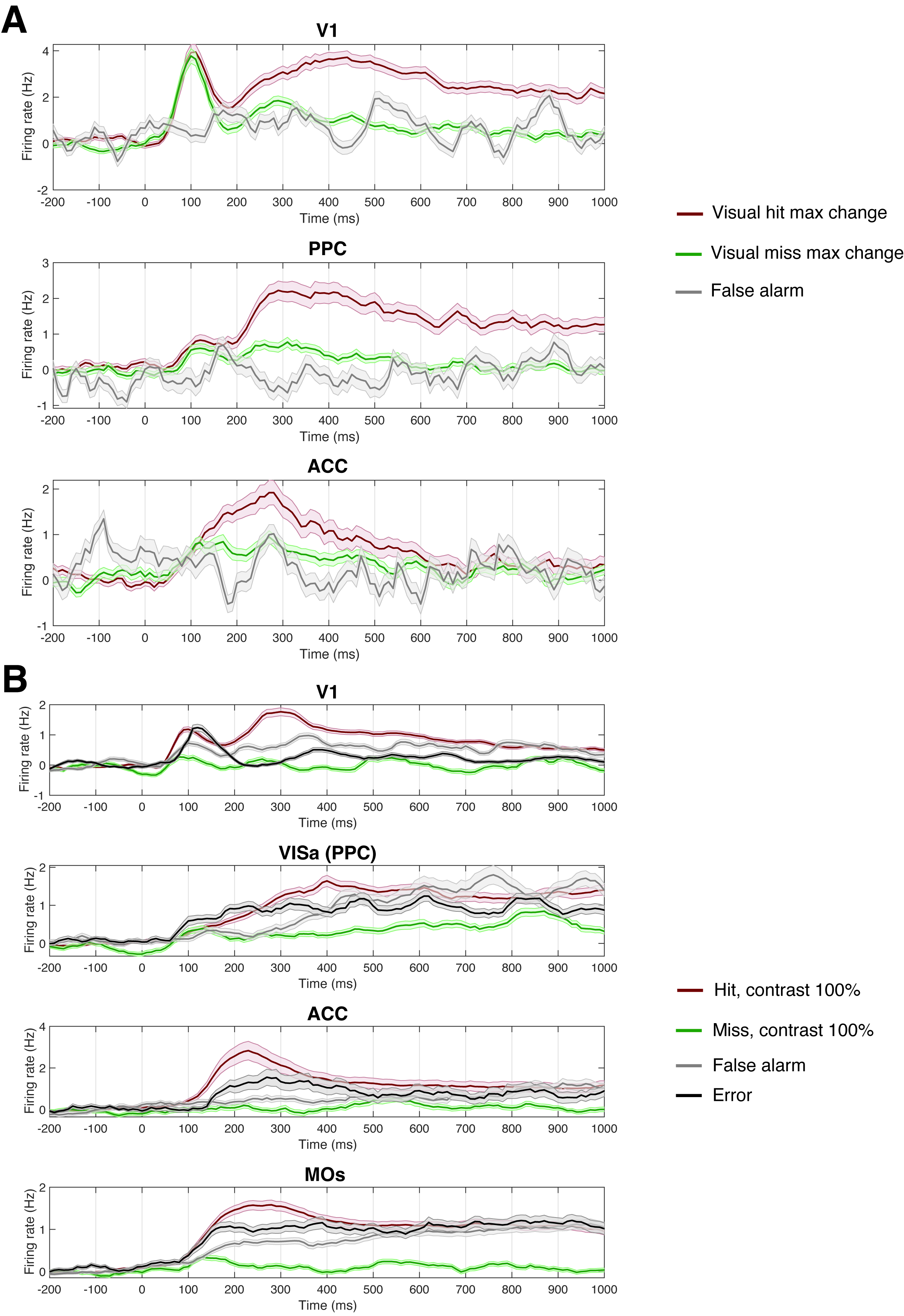

Supplement: Figure 1-1 — (A) Same as Fig. 1C, but including neuronal responses during false alarms (grey). Neuronal responses during error trials (licks toward the wrong side) are not shown in view of their rare occurrence. For visualization purposes, hit and miss trials are only shown for max visual change trials. (B) Same as Fig. 1E, but including neuronal responses during false alarms (grey) and during errors (black), the latter indicating responses towards the lowest-contrast visual stimulus. For visualization purposes, hit and miss trials are only shown for max visual change trials. Download Figure 1-1, TIF file. [file eneuro-11-ENEURO.0191-24.2024-s002.tif]

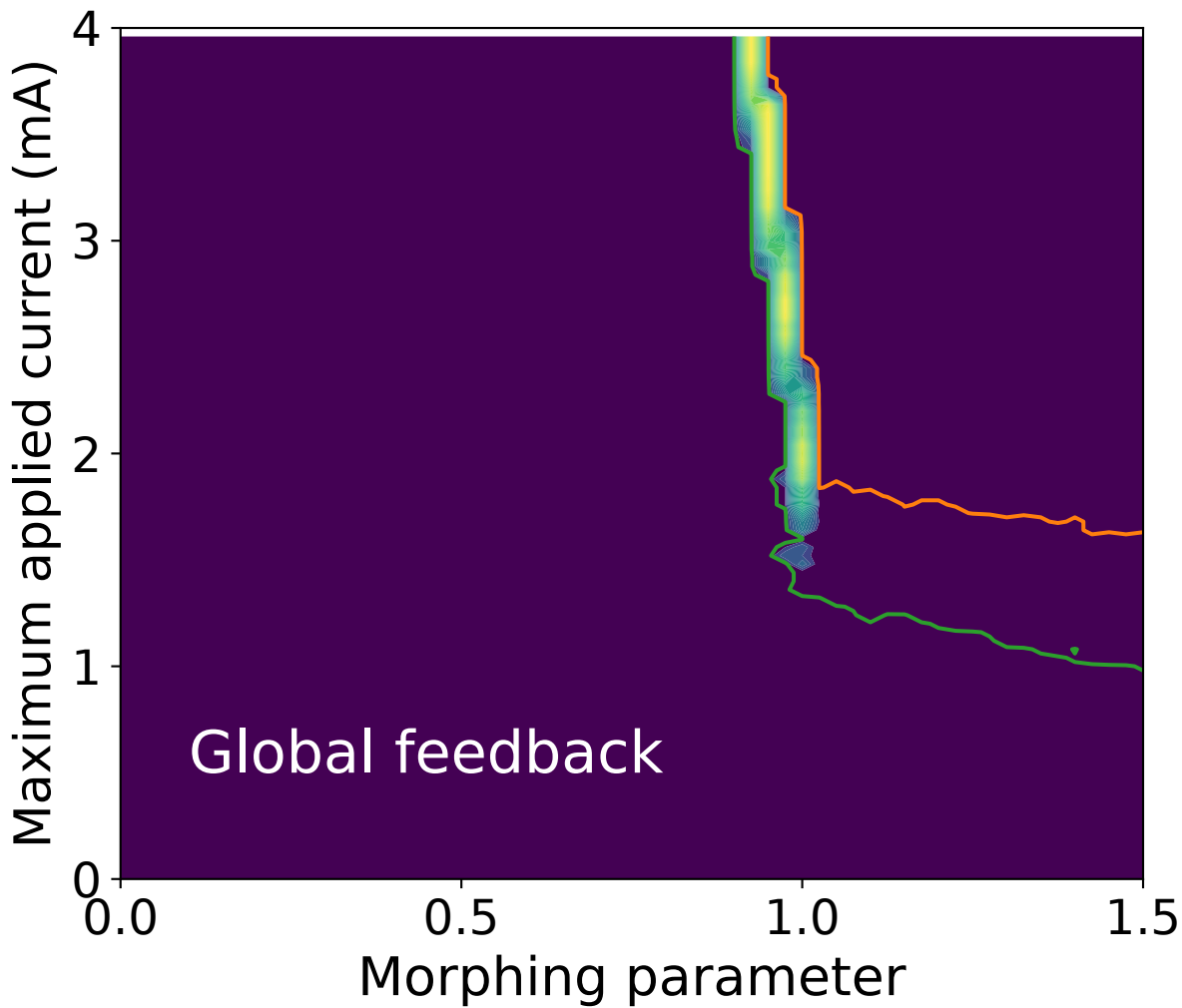

Supplement: Extended Data 1 — The file code.zip includes all code used to generate the figures and perform the analyses presented in this manuscript. The folder "./ephys analysis/" contains code and instructions concerning experimental data and Figure 1. The files "Figure2CDE.m", "Figure3AB.m" and "Figure3C.m" simulate the model in the nominal setup, and generate the corresponding figures. The file "Morphing.m" allows changing the connectivity strengths with respect to the nominal setup, and plotting the resulting trajectories. The folder "./Figure4/" contains instructions, code and data concerning Figure 4. Download Extended Data 1, ZIP file. [file eneuro-11-ENEURO.0191-24.2024-s003.zip › code/Figure4/subfigures/globalfeedback.pdf]

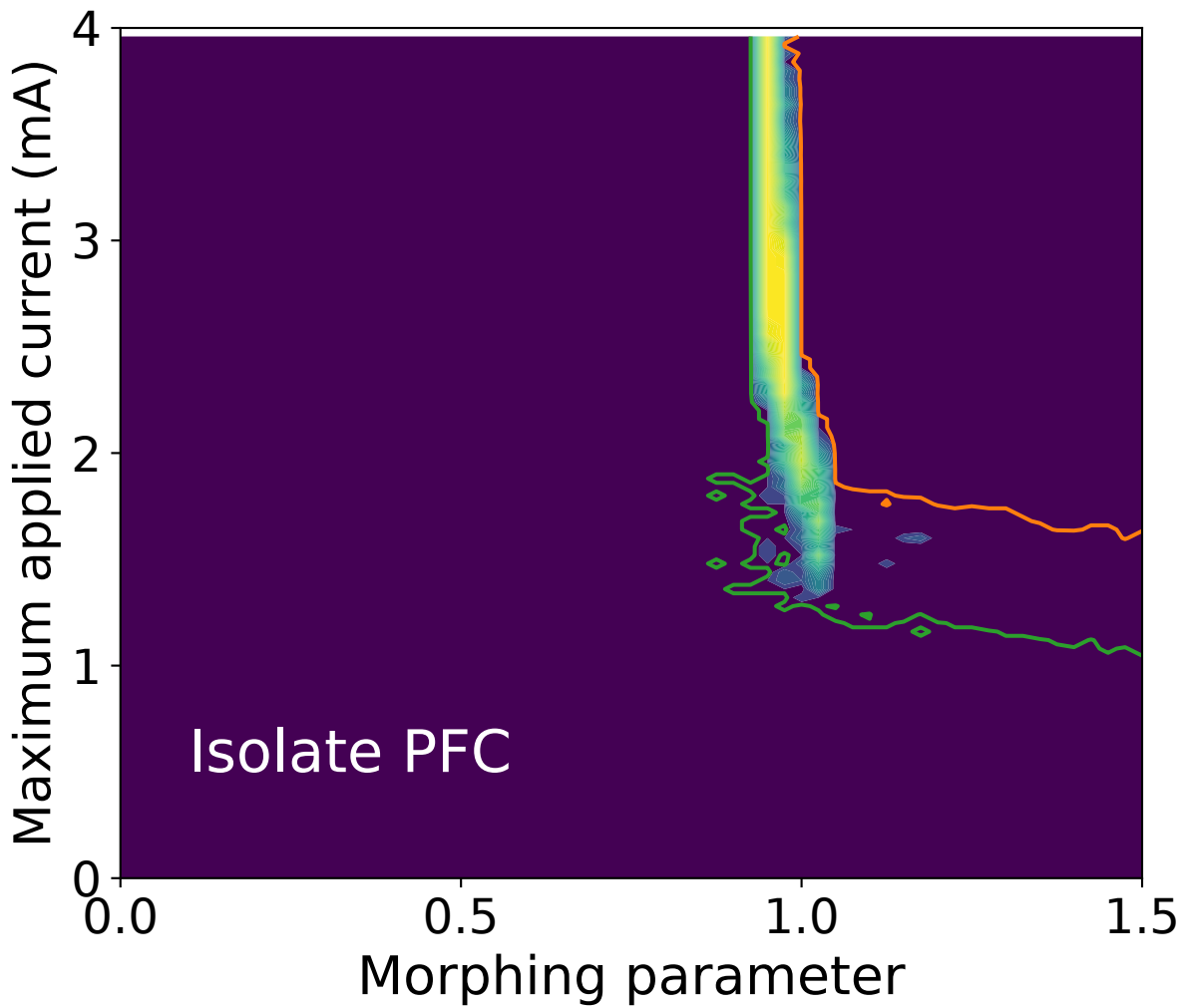

Supplement: Extended Data 1 — The file code.zip includes all code used to generate the figures and perform the analyses presented in this manuscript. The folder "./ephys analysis/" contains code and instructions concerning experimental data and Figure 1. The files "Figure2CDE.m", "Figure3AB.m" and "Figure3C.m" simulate the model in the nominal setup, and generate the corresponding figures. The file "Morphing.m" allows changing the connectivity strengths with respect to the nominal setup, and plotting the resulting trajectories. The folder "./Figure4/" contains instructions, code and data concerning Figure 4. Download Extended Data 1, ZIP file. [file eneuro-11-ENEURO.0191-24.2024-s003.zip › code/Figure4/subfigures/isolatePFC.pdf]
