# Supplementary material for: The Neural and Computational Architecture of Feedback Dynamics in Mouse Cortex during Stimulus Report
Source: eNeuro. 2024 Sep 24;11(9):ENEURO.0191-24.2024. doi: 10.1523/ENEURO.0191-24.2024 (PMC11444237; doi:10.1523/ENEURO.0191-24.2024)
Supplement: Extended Data 1 — The file code.zip includes all code used to generate the figures and perform the analyses presented in this manuscript. The folder "./ephys analysis/" contains code and instructions concerning experimental data and Figure 1. The files "Figure2CDE.m", "Figure3AB.m" and "Figure3C.m" simulate the model in the nominal setup, and generate the corresponding figures. The file "Morphing.m" allows changing the connectivity strengths with respect to the nominal setup, and plotting the resulting trajectories. The folder "./Figure4/" contains instructions, code and data concerning Figure 4. Download Extended Data 1, ZIP file. [file eneuro-11-ENEURO.0191-24.2024-s003.zip › code/Figure4/subfigures/w12.pdf]

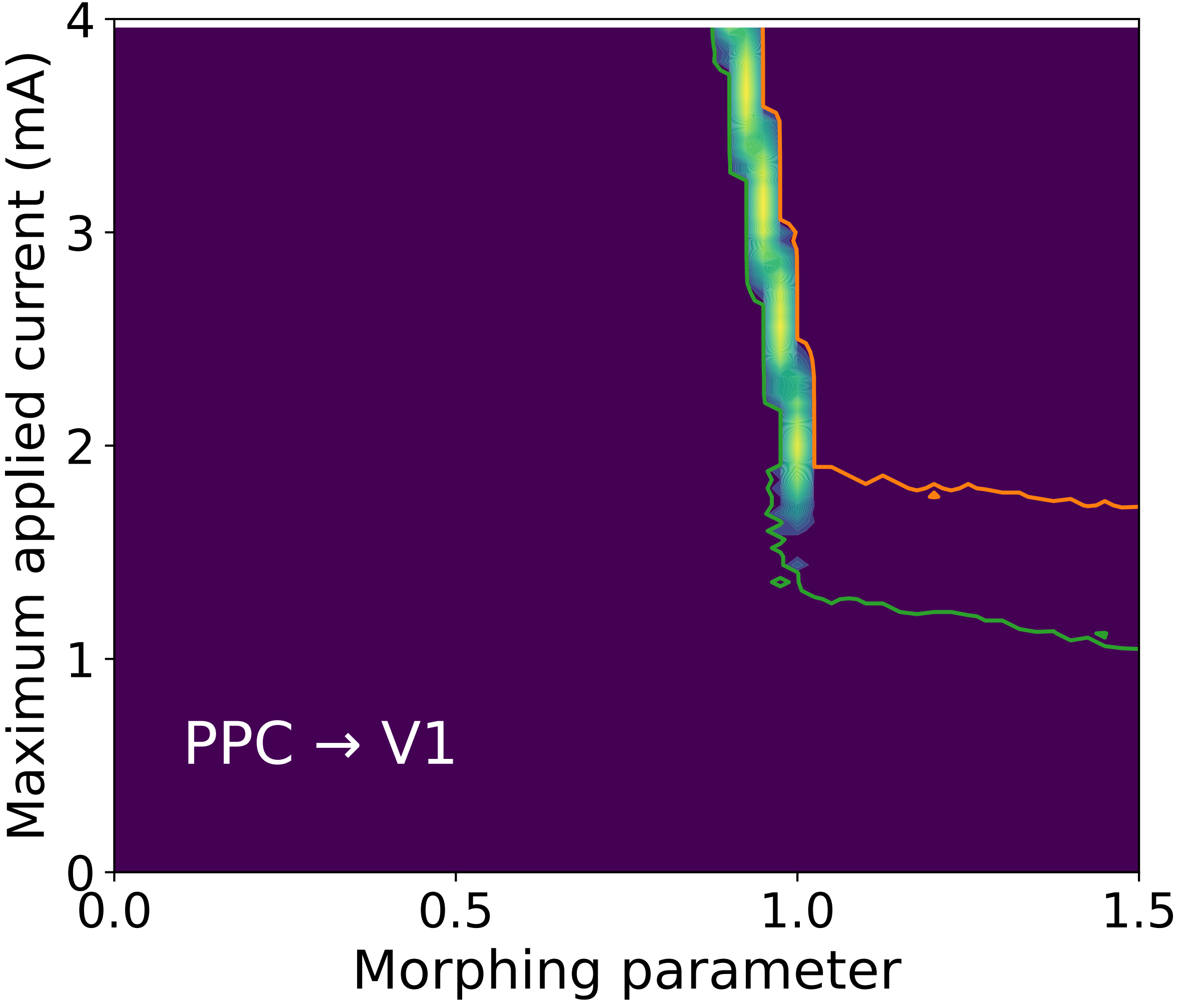

PPC → V1
Maximum applied current (mA)
Morphing parameter
0
1
2
3
4
0.0
0.5
1.0
1.5
